# PAICS mediates DNA damage and cerebellar neuronal loss in C9orf72 amyotrophic lateral sclerosis

**DOI:** 10.1093/brain/awag092

**Published:** 2026-03-11

**Authors:** Jaskaran Singh, Léa Lescouzères, Charlotte Zaouter, Mathilde Chaineau, Ghazal Haghi, Thomas M Durcan, Shunmoogum A Patten

**Affiliations:** Institut National de la Recherche Scientifique (INRS), Centre Armand Frappier Santé Biotechnologie, Laval, QC H7V 1B7, Canada; Institut National de la Recherche Scientifique (INRS), Centre Armand Frappier Santé Biotechnologie, Laval, QC H7V 1B7, Canada; Institut National de la Recherche Scientifique (INRS), Centre Armand Frappier Santé Biotechnologie, Laval, QC H7V 1B7, Canada; Early Drug Discovery Unit (EDDU), The Neuro-Montreal Neurological Institute and Hospital, McGill University, Montreal, QC H3A 1A1, Canada; Early Drug Discovery Unit (EDDU), The Neuro-Montreal Neurological Institute and Hospital, McGill University, Montreal, QC H3A 1A1, Canada; Early Drug Discovery Unit (EDDU), The Neuro-Montreal Neurological Institute and Hospital, McGill University, Montreal, QC H3A 1A1, Canada; Institut National de la Recherche Scientifique (INRS), Centre Armand Frappier Santé Biotechnologie, Laval, QC H7V 1B7, Canada; Département de Neurosciences, Université de Montréal, Montréal, QC H3C 3J7, Canada

**Keywords:** amyotrophic lateral sclerosis, frontotemporal dementia, cerebellum, C9orf72, DNA damage, DNA repair, PAICS

## Abstract

A hexanucleotide (GGGGCC) repeat expansion in the *C9orf72* gene represents the most frequent genetic cause of amyotrophic lateral sclerosis (ALS) and frontotemporal dementia (FTD), resulting in reduced *C9orf72* mRNA and protein expression. *C9orf72* is highly expressed in the cerebellum, and growing evidence implicates C9orf72-associated cerebellar pathology across neurodegenerative disorders, including ALS/FTD, yet the pathogenic mechanisms remain unresolved.

Here, we demonstrate that *in vivo* C9orf72 loss of function leads to cerebellar atrophy, loss of GABAergic interneurons and depletion of Purkinje and granule cells. Additionally, we demonstrate that these cerebellar anomalies precede motor defects. Single-cell transcriptomics of the C9orf72 zebrafish brain revealed the downregulation of a purine biosynthetic gene, *paics*, in Purkinje cells.

Furthermore, we demonstrate the reduced expression of PAICS in human post-mortem cerebellar sections and induced pluripotent stem cell-derived motor neurons from *C9orf72* and sporadic ALS patients. Knockout of *paics* in zebrafish recapitulates cerebellar neuronal loss, neuromuscular junction disruption, motor impairment and widespread DNA damage and repair defects, including suppression of key DNA repair pathways.

Restoring *paics* expression in C9orf72 zebrafish resolves DNA damage and preserves Purkinje cells and granule cells, revealing PAICS as a crucial mediator of cerebellar degeneration and a promising therapeutic avenue for C9orf72-associated ALS and FTD.

## Teaser

Singh *et al.* show that C9orf72 deficiency causes cerebellar degeneration that precedes motor symptoms in ALS. Downregulation of the purine biosynthetic enzyme Paics causes this loss of cerebellar neurons via the accumulation of DNA damage. Restoring *paics* expression reduces DNA damage and rescues cerebellar pathology.

## Introduction

Amyotrophic lateral sclerosis (ALS) is a progressive neurodegenerative disorder characterized by the selective loss of motor neurons that leads to muscular atrophy, paralysis and ultimately death within 2–5 years of the clinical onset of symptoms.^1-3^ The hexanucleotide repeat expansion (HRE) of GGGGCC in the first intronic region of the *C9orf72* gene represents the most common genetic cause of ALS and frontotemporal dementia (FTD).^4-6^ The HRE in the *C9orf72* gene accounts for ∼40% of familial and 7% of sporadic cases of ALS.^7^ HRE drives the pathogenesis of the disease through three distinct mechanisms: (i) the formation of toxic nuclear RNA foci through bidirectional transcription of the expanded repeats^8-10^; (ii) the accumulation of dipeptide repeat proteins generated by repeat-associated non-AUG (RAN) translation^11-13^; and (iii) haploinsufficiency resulting in C9orf72 protein loss-of-function (LOF).^5,6,14^ Although gain-of-function mechanisms are well reported and studied,^15-19^ emerging evidence indicates that C9orf72 haploinsufficiency contributes significantly to the pathogenesis of ALS.^14,20-22^

Although motor neuron degeneration remains the primary hallmark of ALS, neuroimaging studies have revealed broader neurodegeneration affecting multiple brain regions.^23,24^ Consequently, the cerebellum, which plays a crucial role in motor coordination, has emerged as a crucial brain area that shows pathological changes in ALS.^25-29^ Functional MRI, voxel-based morphometry and diffusion-tensor imaging have revealed both functional and structural cerebellar changes in patients with ALS, supporting the characterization of ALS as a multi-system neurodegenerative disorder.^26-28,30,31^ Nonetheless, the molecular mechanisms behind these changes remain poorly understood. C9orf72 is highly expressed in the cerebellum.^6,32^ Additionally, *C9orf72* RNA foci and dipeptide repeat proteins are reported to be present extensively in the cerebellum.^29^ Neuroimaging studies in ALS patients carrying *C9orf72* repeat expansions display significant cerebellar atrophy.^33-35^ Notably, the characteristics of the HRE in the *C9orf72* gene are correlated with clinical and pathological aspects of the disease in the cerebellum.^36,37^ Impairment of cerebellar-specific tasks in C9orf72 patients has been reported.^38^ Moreover, reduced levels of *C9orf72* transcript variants^32,39,40^ and C9orf72 protein in the cerebellum have been reported in *C9orf72* repeat expansion carriers compared with healthy controls.^41,42^ Interestingly, there is a significant association between survival and expression level of the *C9orf72* transcript in the cerebellum.^40^ A recent study has demonstrated focal cerebellar degeneration and disruptions in cerebro-cerebellar connectivity in patients with ALS, which might contribute to both motor and non-motor symptoms of the disease.^35^ Emerging evidence suggests abnormalities in inhibitory GABAergic neurons, particularly the loss of Purkinje cells (PCs), and synapses in ALS.^43,44^ Given the role of the cerebellum in motor coordination and the evidence of its involvement in ALS pathology, studying cerebellar changes in ALS is crucial.

Given that the precise role and mechanism of cerebellar change in ALS remain elusive, we address this gap by investigating the impact of C9orf72 LOF on cerebellar pathology using the zebrafish as a model organism. In this study, we used a stable zebrafish transgenic line with *c9orf72* LOF (C9-*mir*), which exhibits the characteristic hallmarks of ALS,^20^ to study the structural and functional changes in the C9-*mir* zebrafish brain, focusing particularly on the cerebellum. The findings demonstrated global atrophy and the loss of GABAergic neurons in C9-*mir* zebrafish brain and showed cerebellar loss of PCs and granule cells (GCs) preceding motor defects. Notably, our single-cell transcriptomic analysis revealed significant negative enrichment of the purine-containing compound biosynthetic process in PCs, with marked downregulation of the *paics* (phosphoribosylaminoimidazole carboxylase/phosphoribosylaminoimidazole succinocarboxamide synthetase) gene. Importantly, we validated the clinical relevance of these findings by demonstrating significant downregulation of PAICS in the cerebellum and induced pluripotent stem cell (iPSC)-derived motor neurons from both sporadic and *C9orf72*-HRE-carrying ALS patients, suggesting a conserved pathogenic mechanism. Furthermore, *paics* downregulation was sufficient to cause PC and GC loss and DNA damage in C9-*mir* zebrafish. Altogether, our findings strongly demonstrate that *paics* is a key mediator of DNA damage pathology in C9orf72-associated ALS, underscoring the potential role of purine metabolism imbalance in promoting DNA damage and repair failure and fostering disease progression.

## Materials and methods

### Ethics statement

Adult and larval zebrafish (*Danio rerio*) were maintained and experimental procedures performed in compliance with the Canadian Council on Animal Care guidelines and approved by the INRS-LNBE ethics committee. All procedures with human material were approved by the Ethical Committees in Human Research (protocols CER-INRS 2026-659/25-851 for INRS, DURCAN_iPSC/2019–5374 and Institutional Review Board study number A03-M19-22A for McGill University).

### Zebrafish

#### Experimental model maintenance and handling

Wild-type (WT) and C9-*mir* adult zebrafish (*Danio rerio*) were maintained at 28°C under a 12 h–12 h light–dark cycle, as previously described.^45^ The C9orf72 zebrafish model (C9-*mir*) was generated using a micro RNA-based gene-silencing approach.^20^ Embryos were raised at 28.5°C and were collected and staged as previously described.^46^ The following transgenic zebrafish lines were used for imaging studies: Tg(*dlx5a/6a*:GFP) and Tg(*vglut2*:RFP).

#### Generation of *paics* F0 knockout zebrafish

Zebrafish *paics* F0 knockout (KO) embryos were generated using the CRISPR-Cas9 system.^47-49^ The target sequences for guide RNAs (gRNAs) were chosen using CHOPCHOP and Integrated DNA Technologies design tools. Three gRNAs targeting three different exons of the *paics* gene were selected based on the tool-based predictions, with higher priority given to those with fewer off-target effects. The gRNAs targeting the *paics* gene were assembled by annealing each of the three CRISPR RNA (crRNA) (Integrated DNA Technologies) to the Alt-R CRISPR-Cas9 trans-activating crRNA (tracrRNA) (Integrated DNA Technologies, Cat. No. 1072532). Each gRNA was then mixed with Cas9 protein (Alt-R S.p. Cas9 Nuclease V3, Integrated DNA Technologies, Cat. No. 1081058) to form a Cas9/gRNA ribonucleoprotein. All three ribonucleoproteins were pooled (cocktail mix of 6 μl), and 1 nl of the mixture was injected into one-cell-stage embryos. High-resolution melting analysis was performed for genotyping the *paics* mutant zebrafish. The sequences of all primer sets are available upon request.

#### Zebrafish immunostaining and confocal imaging

Zebrafish larvae/sections were fixed in 4% paraformaldehyde (PFA) in PBS at 4°C overnight. The next day, the fish were rinsed three times in a solution of 0.1% Tween-20 in PBS (PBS-Tween) for 15 min at room temperature (RT). Larvae were then rinsed twice with PBS-DT [1% bovine serum albumin (BSA), 1% Triton X-100 and 1% dimethyl sulfoxide (DMSO)] for 10 min each time. Antigen retrieval was performed for zebrafish brain sections at 85°C with 10 mM sodium citrate buffer for 20 min. Larvae/sections were then incubated in a blocking solution [5% normal goat serum (NGS), 1% BSA, 1% DMSO and 1% Triton X-100 in PBS] for 1 h. Primary antibodies [anti-pvalb7 (1:200; Millipore Sigma, Cat. No. MAB1572), anti-neurod1^50^ (gift from M. Hibi; 1:500) and anti-γ-H2AX (1:500; GeneTex, Cat. No. GTX127342)] were added to the blocking solution and incubated overnight at 4°C. The following day, after three 15 min-washes in PBS-Tween, larvae were incubated with a secondary antibody diluted in blocking solution [goat anti-mouse Alexa Fluor 488 (Invitrogen, Cat. No. A10680) or goat anti-rabbit Alexa Fluor 568 (Invitrogen)] at 4°C overnight. The next day, the fish were rinsed three times with PBS-Tween at RT, for 15 min each time. The larvae were mounted the next day in 0.5% low-melting point agarose (Invitrogen, Cat. No. 16520-100) or fluoromount-G (Invitrogen, Cat. No. 00-4958-02) and imaged using a Zeiss LSM 780 confocal microscope.

#### Neuromuscular junction staining and analysis

Zebrafish larvae at 5 days postfertilization (dpf) were fixed overnight at 4°C in 4% paraformaldehyde in PBS. After three 15 min rinses in PBS-Tween at RT, larvae were permeabilized in a collagenase solution (1 mg/ml in PBS; Sigma-Aldrich, Cat. No. C0130) for 2.5 h at RT on a rotator. After three 15 min rinses with PBS-Tween, larvae were blocked for 1 h at RT in a blocking solution (1% BSA, 1% DMSO, 1% Triton X-100 and 2% NGS in PBS). Larvae were incubated for 30 min in α-BTX (tetramethylrhodamine-conjugated α-bungarotoxin; 10 mg/ml in 0.1% PBS-Tween; ThermoFisher, Cat. No. T1175) to label postsynaptic acetylcholine receptors. Then, the primary antibody SV2 (1:200; Developmental Studies Hybridoma Bank) was applied overnight at 4°C in blocking solution. After three 15 min PBS-Tween rinses, larvae were incubated for 4 h at RT in Alexa Fluor 488 goat anti-mouse secondary antibody (1:1000; Invitrogen, Cat. No. A10680) in blocking buffer on a rotator. Larvae were rinsed three times in PBS-Tween and mounted in fluoromount-G (Invitrogen, Cat. No. 00-4958-02). Confocal imaging was performed using a Zeiss LSM 780 microscope. The analysis was performed via NMJ Analyser using the default parameter of zebrafish NMJ.^51^

### Single-cell RNA sequencing

#### Library preparation and sequencing

Single-cell RNA sequencing (scRNA-seq) libraries were prepared from dissociated adult C9*-mir* and WT zebrafish brains using standard protocols for cell capture, complementary DNA synthesis and library preparation, followed by sequencing on an Illumina NovaSeq 6000 platform (for detailed methodology, see the Supplementary material).

#### Data analysis

Single-cell RNAseq data were processed and analysed using Seurat (v.5) and DoubletFinder in R for rigorous quality control, doublet exclusion, dimensional reduction and cell type annotation. Subsequent differential gene expression and gene set enrichment analysis (GSEA) were performed to identify transcriptomic alterations and dysregulated pathways in specific cerebellar populations, particularly PCs and GCs. Detailed bioinformatics pipelines, parameters and filtering thresholds are provided in the Supplementary material.

### Induced pluripotent stem cell-derived motor neurons

#### Cell lines and reprogramming

Peripheral blood mononuclear cells (PBMCs) were obtained through the C-BIG Repository at the Montreal Neurological Institute (The Neuro, McGill University). Three cells lines were used, which were derived from a patient carrying a *C9orf72*-HRE and a patient with sporadic ALS, reprogrammed from PBMCs of a 66-year-old male and a 65-year-old female, respectively, and a previously published and characterized control cell line, AIW002-02,^52^ reprogrammed from PBMCs of a 37-year-old Caucasian male. Differentiation of iPSCs into motor neurons was performed using a previously published protocol.^53^ The iPSC-derived motor neurons were cultured and collected for two independent studies, including this one. The detailed methodology is described by Lescouzères *et al*.^54^ Briefly, iPSCs were initially induced into neuroepithelial progenitors, which were next specified into motor neuron progenitor cells over 6 days. The motor neuron progenitor cells were subsequently replated for terminal differentiation into mature motor neurons. These were cultured for 14 and 28 days post-plating to capture two complementary maturation stages, representing early post-mitotic motor neurons with initial axon extension and mature motor neurons, respectively. Differentiation efficiency was confirmed by immunofluorescence and qPCR.^54^ Expression of the mature motor neuron markers HB9 (MNX1; Supplementary Fig. 1), ISL1 and CHAT was strongly upregulated in mature motor neurons, whereas it was nearly absent in the iPSCs and motor neuron progenitor cells.^54^

### Human post-mortem tissues

#### Cerebellum samples

Human cerebellum samples were acquired from the Target ALS Multicenter Human Postmortem Tissue Core. Fixed, paraffin-embedded sections of cerebellum were obtained from three patients with C9orf72 ALS, a sporadic case and three non-neurological controls. Samples were from a mixture of males and females.

#### Extended experimental procedures

Detailed protocols regarding zebrafish *in vitro* transcription, morphological and behavioural assessments, histological analyses (haematoxylin and eosin, TUNEL and immunostaining) and molecular assays (qPCR and western blotting) are provided in the Supplementary material. Additionally, comprehensive methodologies for immunostaining, western blotting and qPCR of iPSC-derived motor neurons, in addition to the processing and immunostaining of human post-mortem cerebellar tissues, are described in the Supplementary material.

### Statistical analysis

Significance was determined using Student’s *t*-test or one-way ANOVA followed by a multiple comparisons test. Graphs were plotted using Graphpad PRISM or custom-made visualization scripts in R. Significance is indicated as *P* < 0.05, *P* < 0.01 or *P* < 0.001; *N* represents the number of experimental repeats, and *n* represents the number of fish. For the iPSC-derived motor neurons, each experiment was performed using technical replicates generated by independent differentiations. Data are presented as the mean ± standard error of the mean.

## Results

### Brain atrophy and loss of GABAergic neurons in C9orf72 loss-of-function zebrafish model

We recently showed that a zebrafish C9orf72 LOF (C9-*mir*) model at 6 days postfertilization (6 dpf) recapitulates ALS phenotypes, including motor deficits, motor neuron loss, muscle atrophy and TDP-43 pathology.^20^ Capitalizing on the strength of this in *vivo* ALS model, we sought to investigate the effect of C9orf72 LOF on brain architecture. We performed histological studies of brain sections from C9-*mir* zebrafish at the larval stage (6 dpf) and the adult stage (12 months postfertilization). Haematoxylin and eosin staining of transverse sections at 6 dpf showed a significant reduction in the size of the C9-*mir* brain along the whole rostrocaudal axis (Fig. 1A and B). Interestingly, we observed weaker haematoxylin staining in the cerebellum of C9-*mir* zebrafish brain compared with controls, suggesting cellular depletion in the region (Fig. 1A). This phenotype persisted in adulthood, as demonstrated by a sagittal section of the adult C9-*mir* zebrafish brain showing a significant reduction in the area of the cerebellum (Fig. 1C and D). Taken together, these findings are in line with clinical observations in ALS-FTD patients, where neuroimaging studies have documented progressive brain atrophy extending beyond motor regions.^23,24,55,56^

**Figure 1:**
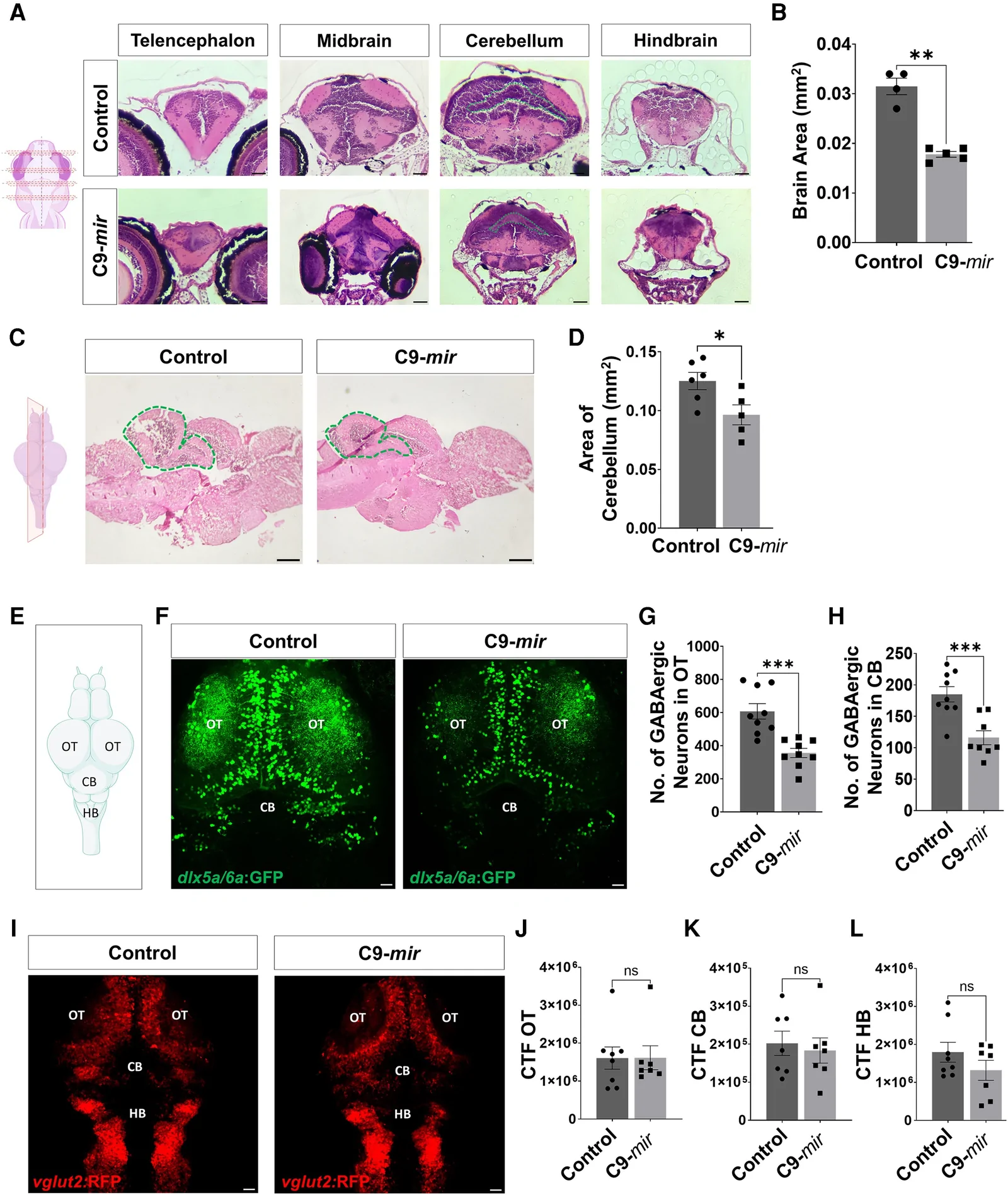
**C9orf72 loss-of-function causes early brain atrophy and loss of GABAergic neurons in zebrafish.** (**A**) Haematoxylin and eosin staining of the telencephalon, midbrain, cerebellum and hindbrain sections of control and C9-*mir* zebrafish at 6 days postfertilization (dpf). Green dashed line indicates the cerebellum. Scale bar = 10 µm. (**B**) Quantitative analysis demonstrated a significant decrease in the brain area of C9-*mir* compared with control zebrafish brain at 6 dpf. *N* = 4 or 5 biological replicates. (**C**) Haematoxylin and eosin staining on a sagittal section of the adult control and C9-*mir* zebrafish brain. Green dashed line indicates the cerebellum. Scale bar = 100 µm. (**D**) Quantitative analysis demonstrated a significant decrease in cerebellar area in C9-*mir* compared with control adult zebrafish. *N* = 5 or 6 biological replicates. (**E**) Schematic representation of the zebrafish brain. (**F**) Representative images of GABAergic neurons (*dlx5a/6a*:GFP) in control versus C9-*mir* zebrafish brain at 5 dpf. Scale bar = 20 µm. (**G**) Quantitative analysis demonstrated a significant decrease in the number of GABAergic neurons (*dlx5a/6a*:GFP) in C9-*mir* compared with control optic tectum at 5 dpf, *n* = 9 samples. (**H**) Quantitative analysis demonstrated a significant decrease in the number of GABAergic neurons (*dlx5a/6a*:GFP) in C9-*mir* compared with control cerebellum at 5 dpf, *n* = 8 or 9 samples. (**I**) Representative images of glutamatergic neurons (*vglut2*:RFP) in control versus C9-*mir* zebrafish brain at 5 dpf. Scale bar = 20 µm. (**J**) Quantitative analysis demonstrated no significant changes in the corrected total fluorescence (CTF) of glutamatergic neurons (*vglut2*:RFP) in C9-*mir* compared with control optic tectum at 5 dpf, *n* = 7 or 8 samples. (**K**) Quantitative analysis demonstrated no significant changes in the CTF of glutamatergic neurons (*vglut2*:RFP) in C9-*mir* compared with control cerebellum at 5 dpf, *n* = 7 or 8 samples. (**L**) Quantitative analysis demonstrated no significant changes in the CTF of glutamatergic neurons (*vglut2*:RFP) in C9-*mir* compared with control hindbrain at 5 dpf, *n* = 7 or 8 samples. Data are mean ± standard error of the mean. Student’s *t*-test; *P < 0.05, **P < 0.01, ***P < 0.001, ****P < 0.0001; ns = non-significant. CB = cerebellum; HB = hindbrain; OT = optic tectum. Figure partially created in BioRender. Patten, K. (2026) https://BioRender.com/a32owax.

Given the brain size and cerebellar abnormalities in C9-*mir* zebrafish, we next conducted an analysis of the brain neuronal networks. We observed a significant reduction in the number of GABAergic neurons in the C9-*mir* zebrafish brain at 5 dpf (Fig. 1E and F), particularly in the optic tectum (Fig. 1F and G) and cerebellum (Fig. 1F and H). These findings align with the emerging clinical evidence of GABAergic system dysfunction in ALS patients.^57-63^ We did not observe any changes in the glutamatergic neurons in the C9-*mir* zebrafish brain at 5 dpf (Fig. 1I–L) or 6 dpf (Supplementary Fig. 1B–E). These data suggest a potential imbalance between excitatory and inhibitory neural networks in the zebrafish C9-*mir* ALS model.

### Loss of Purkinje cells and granule cells in the cerebellum precedes motor defects in C9-*mir* zebrafish

To complement our previous observations of cerebellar abnormalities in C9-*mir* zebrafish and explore cerebellar cell populations further, using immunostaining, we assessed two main cerebellar neuronal cell types: parvalbumin7 (pvalb7)-positive PCs and neurod1-positive GCs, both integral to cerebellar function.

At 5 dpf, we observed a significant reduction in the numbers of PCs and GCs within the cerebellum of C9-*mir* zebrafish (Fig. 2A–C). This neuronal loss persisted into adulthood, as depicted by the sagittal sections of the adult C9-*mir* zebrafish cerebellum, which continued to show a marked decrease in the number of PCs (Fig. 2D and E). The cerebellum plays a central role in the coordination of motor functions, such as swimming behaviour, in larval zebrafish.^64,65^ We thus investigated the temporal relationship between cerebellar pathology and motor dysfunction in the C9-*mir* zebrafish using the automated Noldus Ethovision XT behaviour monitoring system. At 5 dpf, the C9-*mir* zebrafish exhibited no significant motor impairments, with both distance travelled and velocity comparable to control fish (Fig. 2F–H). However, by 6 dpf, the C9-*mir* zebrafish displayed significant motor deficits, characterized by reduced distance travelled and decreased velocity (Fig. 2I–K). These findings indicate that loss of PCs and GCs in C9-mir zebrafish cerebellum precedes motor dysfunction, thus emphasizing the importance of early cerebellar involvement in the pathogenesis of ALS.

**Figure 2:**
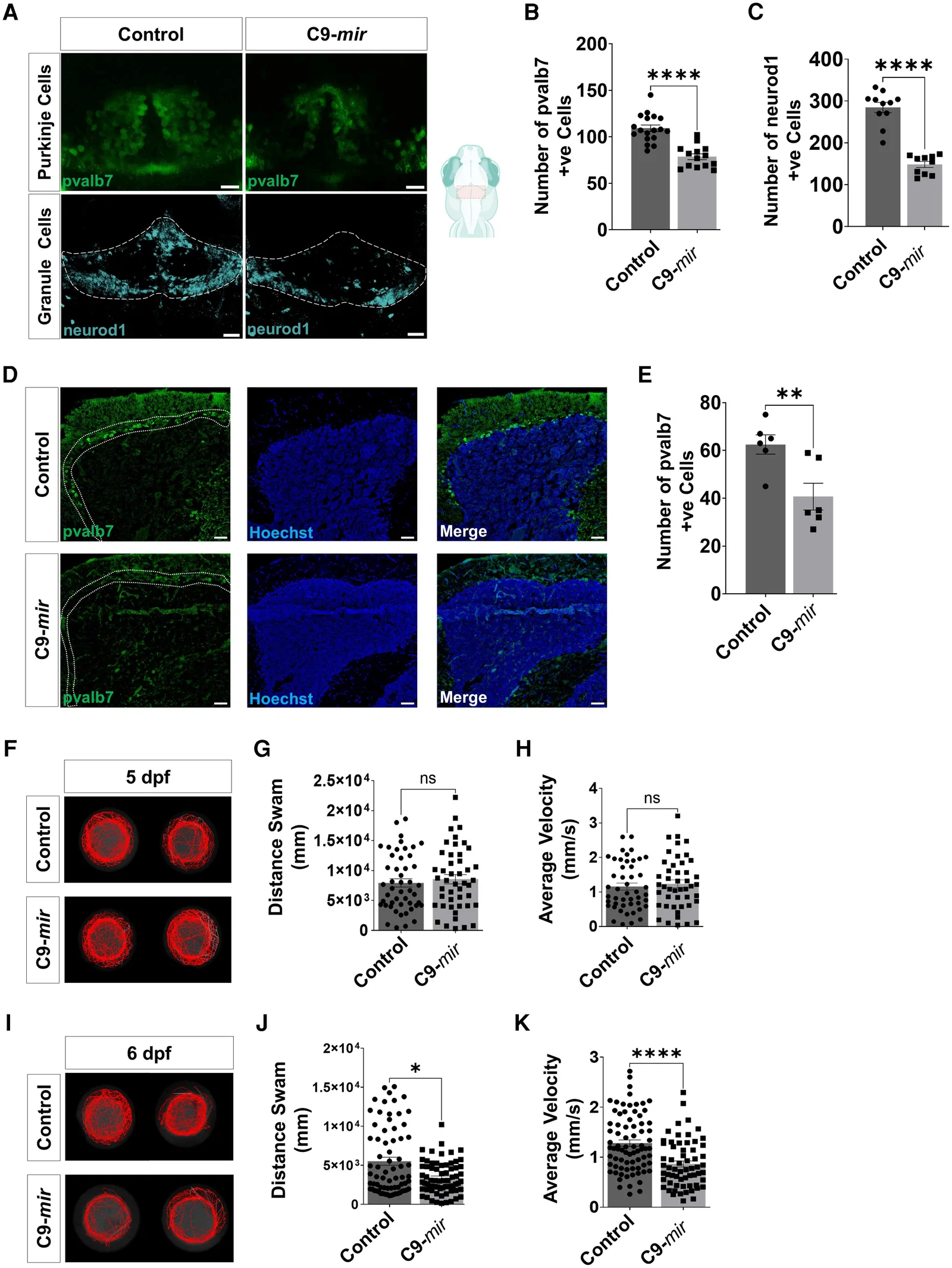
**Purkinje and granule cell loss precedes the onset of motor impairment in C9-*mir* zebrafish.** (**A**) Immunostaining of Purkinje cells (pvalb7) and granule cells (neurod1) in control and C9*-mir* zebrafish cerebellum at 5 days postfertilization (dpf). Scale bar = 10 µm for Purkinje cells and 20 µm for granule cells. (**B**) Quantitative analysis demonstrated a significant decrease in the number of Purkinje cells (pvalb7) in C9-*mir* zebrafish compared with control zebrafish cerebellum at 5 dpf, *n* = 14–18. (**C**) Quantitative analysis demonstrated a significant decrease in the number of granule cells (neurod1) in C9-*mir* zebrafish compared with control zebrafish cerebellum at 5 dpf, *n* = 10 or 11. (**D**) Sagittal section of adult zebrafish cerebellum in control and C9-*mir* zebrafish. White dashed lines indicate the cell bodies of Purkinje cells. Scale bar = 50 µm. (**E**) Quantitative analysis demonstrated a significant decrease in the number of Purkinje cells (pvalb7) in adult C9-*mir* zebrafish compared with control zebrafish section of cerebellum, *n* = 6. (**F**) Representative swimming tracks of control and C9-*mir* zebrafish at 5 dpf. (**G** and **H**) The C9-*mir* zebrafish (*n* = 46) did not show any significant changes in swimming distance (**G**) and average velocity (**H**) compared with control zebrafish (*n* = 47–48) at 5 dpf. (**I**) Representative swimming tracks of control and C9-*mir* zebrafish at 6 dpf. (**J** and **K**) The C9-*mir* zebrafish (*n* = 62–69) displayed a significant decrease in swimming distance (**J**) and average velocity (**K**) compared with control zebrafish (*n* = 71–77) at 6 dpf. All data are represented as the mean ± standard error of the mean. Statistical significance was calculated by Student’s *t*-test. ns = non-significant; *P < 0.05, **P < 0.01, ***P < 0.001, ****P < 0.0001. Figure partially created in BioRender. Patten, K. (2026) https://BioRender.com/9maheqa.

### Dysregulated purine-containing compound biosynthesis process in C9*-mir* Purkinje cells

To elucidate the mechanistic link between the loss of PCs and GCs and the pathogenesis of ALS, we performed scRNA-seq on the adult C9-*mir* and WT control zebrafish brain tissue. The data were processed using the Seurat standard pipeline for data filtering and clustering to determine cell type identity based on genetic markers of zebrafish brain cell populations.^60,61^ We identified 15 major cell clusters within the zebrafish brain (Fig. 3A), each characterized by unique genetic markers (Fig. 3B). We used the zebrafish cell landscape,^66^ zebrahub,^67,68^ the single-cell transcriptome atlas for zebrafish^69-71^ and the Max Planck zebrafish brain (mapzebrain) atlas^72^ as major reference databases of genetic markers for cell annotations. Given that PCs did not appear in the 15 major cell clusters, we specifically isolated a cluster of PCs using carbonic anhydrase 8 (*ca8*) as a genetic marker.^73^ Furthermore, we cross-referenced the top 10 differentially expressed genes (DEGs) in each of the identified cell clusters against publicly available ALS studies.^74-78^ Notably, this comparison revealed that a substantial number of these genes have previously been associated with ALS in the literature (Supplementary Table 1), providing strong validation for our data.

**Figure 3:**
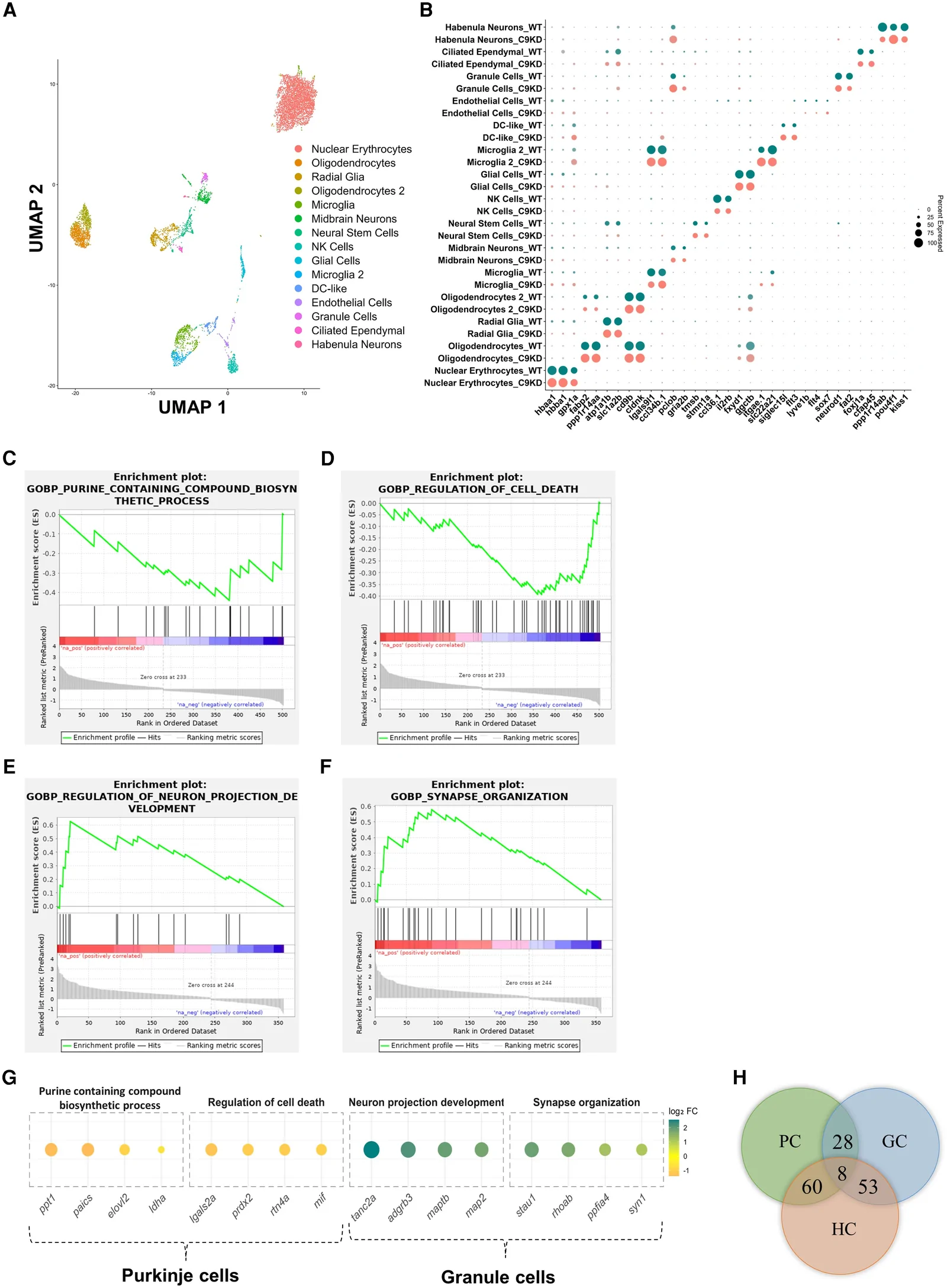
**Comprehensive analysis of single-cell RNA sequencing and differential gene expression in adult wild-type and C9-*mir* zebrafish brain**. (**A**) Uniform manifold approximation and projection (UMAP) of single-cell RNA sequencing profiles from adult wild-type (WT) and C9-*mir* zebrafish brains, representing 15 cell clusters. (**B**) Dot plot representing two or three marker genes for each cell cluster in the UMAP. (**C**–**F**) Enrichment plots from gene set enrichment analysis representing top enriched biological processes from Purkinje cells (PCs) and granule cells (GCs) in C9-*mir* zebrafish cerebellum. (**G**) Dot plot representing the top differentially expressed genes (DEGs) from the enriched biological processes in PCs and GCs. (**H**) Venn diagram illustrating the overlap of DEGs between human cerebellar tissue from *C9orf72*-HRE amyotrophic lateral sclerosis patients and C9-*mir* zebrafish cerebellar cell types. *Right*: Fifty-three shared DEGs between human cerebellum (HC) and GCs. *Left*: Sixty shared DEGs between HC and PCs.

Next, we performed a GSEA on the DEGs in PCs and GCs, respectively, to identify dysregulated biological processes in C9-*mir* zebrafish. GSEA offers the advantage of assessing gene expression data at the level of gene sets, making it particularly capable of detecting slight, coordinated changes in biological pathways that otherwise could be missed when focusing solely on individual genes.^79,80^ GSEA on the DEGs in PCs revealed significant negative enrichment in pathways related to the nucleoside biosynthetic process (Supplementary Fig. 2A), purine-containing compound biosynthetic process (Fig. 3C and Supplementary Fig. 2A) and regulation and negative regulation of cell death in the C9-*mir* PCs (Fig. 3D and Supplementary Fig. 2A). This suggests a downregulation of genes involved in purine nucleotide synthesis and cell survival mechanisms, most notably *paics* and *rtn4a* (Fig. 3G and Supplementary Table 2), potentially compromising neuronal function and viability. Additionally, pathways related to neuron projection development (Fig. 3E and Supplementary Fig. 2B), synapse organization (Fig. 3F and Supplementary Fig. 2B) and autophagy (Supplementary Fig. 2B) were found to be dysregulated in the C9-*mir* GCs, indicating alterations in genes essential for neuronal connectivity, synaptic integrity and cellular maintenance (Fig. 3G and Supplementary Table 2), which might contribute to cerebellar dysfunction.

To further validate the translational relevance of our findings, we compared our single-cell transcriptomic data from C9-*mir* zebrafish with published human cerebellar transcriptome data from *C9orf72*-HRE-carrying ALS patients.^81^ This cross-species analysis revealed remarkable overlap in DEGs. Specifically, 53 DEGs were shared among human cerebellar tissue and GCs in our zebrafish dataset, and 60 DEGs were shared among human cerebellar tissue and PCs (Fig. 3H). These overlapping genes, particularly in cerebellar cell types, represent robust clinical validation of our zebrafish model as a reliable system for studying ALS-related molecular mechanisms.

### Conserved PAICS downregulation in C9*-mir* zebrafish and *C9orf72*-ALS patient samples

Recent studies have underlined the importance of purine metabolism in neurodegenerative diseases, including ALS.^82,83^ Our single-cell RNA data revealed significant downregulation of the *paics* gene, which encodes an enzyme essential for *de novo* purine biosynthesis, in PCs of the C9-*mir* zebrafish cerebellum (Fig. 4A and Supplementary Fig. 3A). Consistent with the transcriptomic changes, the level of Paics protein was also significantly reduced in C9-*mir* zebrafish, as determined by western blot analysis (Fig. 4B and C). To explore whether PAICS might interact physically with C9orf72, we modelled a PAICS–C9orf72 complex using AlphaFold 3^84^ (DeepMind). The individual protein structures were predicted with high confidence, but a low interface predicted template modelling score (ipTM = 0.32) and diffuse inter-chain predicted aligned error indicated that AlphaFold does not support a stable and well-defined PAICS–C9orf72 complex (Supplementary Fig. 3B). These *in silico* results from AlphaFold 3, therefore, argue against a strong direct interaction and hint at an indirect mechanism linking PAICS to C9orf72. Further analysis of *paics* expression across 14 distinct cell types in the C9-*mir* zebrafish brain revealed widespread downregulation in 9 of these cell types (Supplementary Fig. 3C).

**Figure 4:**
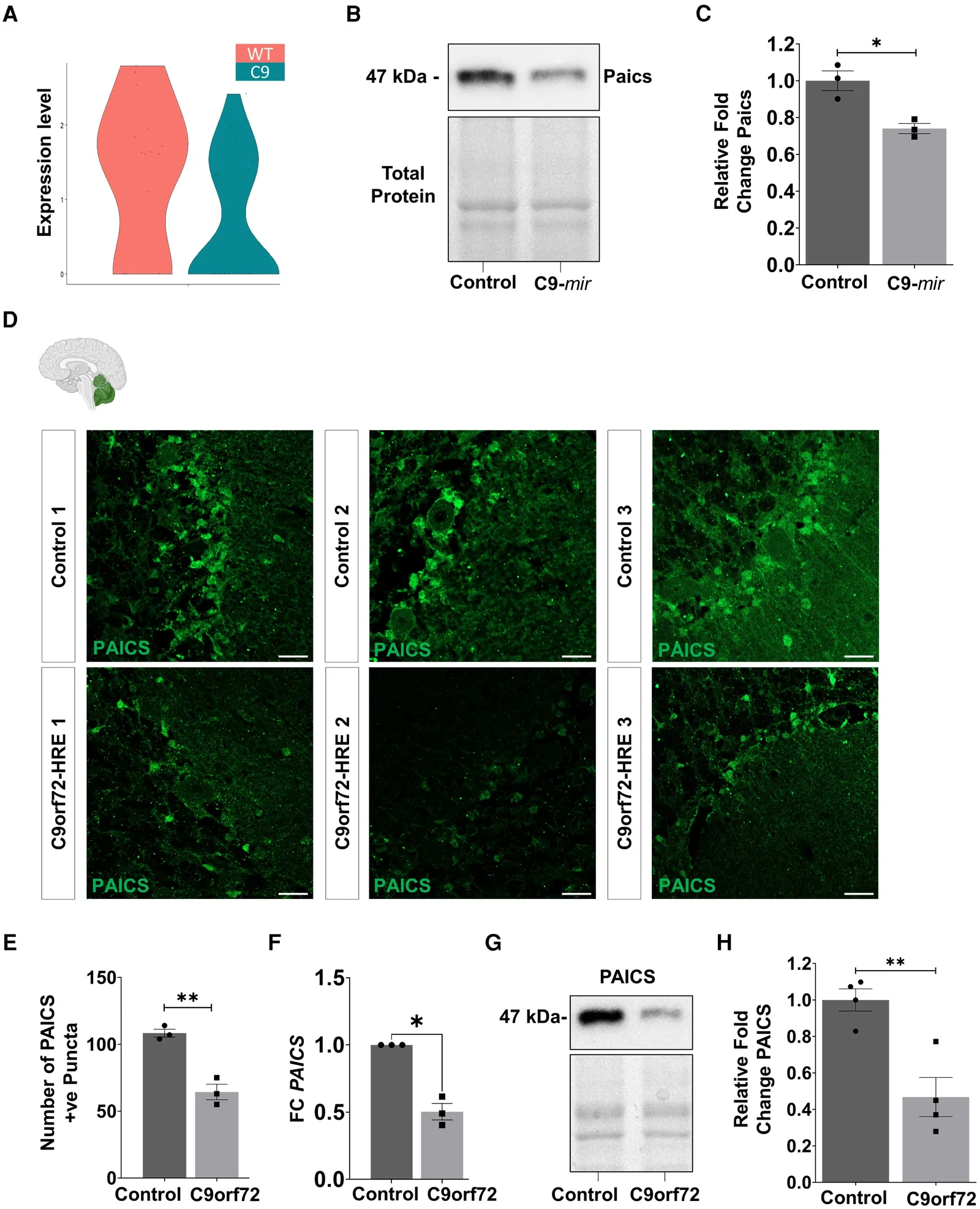
**PAICS expression is downregulated in C9-*mir* zebrafish, human post-mortem cerebellum sections and induced pluripotent stem cell-derived motor neurons from patients with amyotrophic lateral sclerosis.** (**A**) Violin plot representing the expression level of *paics* gene in C9-*mir* versus wild-type (WT) Purkinje cells. (**B**) Western blot depicting the level of Paics protein in control and C9-*mir* zebrafish at 5 days postfertilization (dpf). (**C**) Quantification of the Paics protein level in control and C9-*mir* zebrafish (*n* = 3) at 5 dpf. (**D**) Immunofluorescence staining of PAICS protein in post-mortem cerebellar sections from control and *C9orf72*-HRE ALS patients. *N* = 3. Scale bar = 30 μm. (**E**) Quantitative analysis demonstrated a significant decrease in the number of PAICS^+^ puncta in *C9orf72*-HRE cerebellum sections compared with controls (*N* = 3). (**F**) Quantitative PCR (qPCR) analysis of *PAICS* mRNA in iPSC-derived motor neurons (MNs) from control and *C9orf72*-HRE ALS patients at 3 weeks post-differentiation. (*N*′ = 3). (**G**) Western blot depicting the level of PAICS protein in iPSC-derived MNs from control and *C9orf72*-HRE ALS patients at 1 week post-differentiation. (**H**) Quantification of the PAICS protein level in iPSC-derived MNs from control and *C9orf72*-HRE ALS patients at 1 week post-differentiation (*N*′ = 4). All data are represented as the mean ± standard error of the mean. Statistical significance was calculated by Student’s one-tailed *t*-test (with Welch’s correction for unequal variances). *P < 0.05, **P < 0.01, ***P < 0.001, ****P < 0.0001. ALS = amyotrophic lateral sclerosis; iPSC = induced pluripotent stem cell; *n* = zebrafish sample size; *N* = number of human donors from whom post-mortem cerebellar tissue was obtained; *N*′ = number of donors from whom iPSC-derived MN lines were generated. Figure partially created in BioRender. Patten, K. (2026) https://BioRender.com/h0zh8h0.

The zebrafish genome encodes a single *paics* orthologue that shares a high degree of similarity with human *PAICS* (71.3% nucleotide and 76.3% amino acid identity). According to the Human Protein Atlas, PAICS is expressed in all tissues, including highly in the brain, particularly the cerebral cortex and cerebellum. To validate the translational significance of Paics downregulation observed in our C9-*mir* zebrafish model, we next examined the PAICS expression in post-mortem cerebellar tissue from three controls and three *C9orf72*-HRE ALS patients. Hoechst staining confirmed the characteristic cerebellar foliation in these samples (Supplementary Fig. 3D). In line with our zebrafish data, we observed a significant reduction in PAICS expression in the cerebellum of *C9orf72*-HRE ALS patients compared with controls (Fig. 4D and E). Notably, the PAICS signal appeared enriched in cells with a morphology and perisomatic distribution consistent with Bergmann glia surrounding Purkinje neuron cell bodies (Supplementary Fig. 3E). Additionally, the examination of cerebellar tissue from one sporadic ALS patient revealed reduced PAICS expression relative to control (Supplementary Fig. 3F), thereby raising the possibility that PAICS downregulation is not restricted to C9orf72-ALS.

Given that we observed a significant loss of GCs in the C9-*mir* zebrafish cerebellum, we next sought to examine the GC population in post-mortem human cerebellar tissue. Mirroring the changes observed in the C9-*mir* zebrafish cerebellum, we observed a significant reduction in the GABA-AR α1 signal, which marks GCs in the cerebellum from three *C9orf72*-HRE ALS patients compared with three controls (Supplementary Fig. 4A and B). Examination of cerebellar tissue from one sporadic ALS patient similarly showed a reduction in GABA-AR α1 signal relative to the control (Supplementary Fig. 4C).

As indicated by our single-cell RNA data, that *paics* downregulation was not restricted to the cerebellum but occurred across 9 of 14 identified cell types in the zebrafish brain (Supplementary Fig. 3C). Moreover, we observed a global reduction in the expression of the Paics protein in C9-*mir* zebrafish (Fig. 4B and C). Given this widespread downregulation and the fact that motor neuron loss is a primary hallmark of ALS, we next examined *PAICS* expression in iPSC-derived motor neurons from *C9orf72*-HRE carriers and sporadic ALS patients. To this end, we generated iPSC-derived motor neurons from *C9orf72*-HRE carriers, sporadic ALS patients and controls using a sequential differentiation protocol (Supplementary Fig. 4D). Quantitative PCR (qPCR) and western blot analysis of iPSC-derived motor neurons from *C9orf72*-HRE patients revealed a significant reduction of PAICS mRNA (Fig. 4F) and protein levels (Fig. 4G and H), respectively, compared with controls. Immunostaining confirmed PAICS expression in motor neurons, but no major difference was observed in the cellular location of PAICS in control versus *C9orf72-*HRE iPSC-derived motor neuron lines (Supplementary Fig. 4E). Additionally, we also observed reduced *PAICS* mRNA expression in iPSC-derived motor neurons from patients with sporadic ALS relative to controls (Supplementary Fig. 4F). Altogether, these findings indicate that downregulation of PAICS expression is a conserved feature across zebrafish, post-mortem cerebellum and patient-derived motor neurons.

### *paics* knockout in zebrafish leads to motor dysfunction

PAICS is a bifunctional enzyme that catalyses sequential steps in the *de novo* purine biosynthesis pathway: the conversion of 5-aminoimidazole ribonucleotide to 5′-phosphoribosyl-4-carboxy-5-aminoimidazole and the subsequent transformation of 5′-phosphoribosyl-4-carboxy-5-aminoimidazole into 5-aminoimidazole-4-(*N*-succinylcarboxamide) ribonucleotide.^85^ These reactions are important in the synthesis of purine nucleotides, the basic components of DNA and RNA, and play a critical role in cellular energy metabolism. Dysregulation in the function of PAICS has been implicated in several pathological conditions, including neurodevelopmental disorders,^86^ highlighting its importance in maintaining cellular homeostasis.

To investigate the functional consequences of *paics* downregulation, we generated a *paics* F0 CRISPR KO zebrafish line. Zebrafish *paics* F0 KO embryos were generated using the CRISPR-Cas9 system.^49^ We targeted three gRNA sites at exons 3, 5 and 6 of the zebrafish *paics* gene to disrupt its function (Supplementary Fig. 5A). This approach facilitated the generation of a biallelic zygotic KO directly in the injected embryos of the F0 generation.^47,48^ A high-resolution melting assay^87^ revealed that the gRNAs were highly efficient in creating indels in injected embryos (Supplementary Fig. 5B).

We observed that *paics* F0 KO fish displayed a significant reduction in head length (Fig. 5A) and head area (Fig. 5A) compared with WT control zebrafish at 5 dpf. Motor behavioural analysis further revealed that *paics* F0 KO zebrafish exhibited notable motor deficits at 5 dpf, with a significant reduction in the distance travelled and velocity of the *paics* F0 KO zebrafish compared with WT control zebrafish at 5 dpf (Fig. 5B). These results were consistent with the motor phenotype observed in our C9-*mir* zebrafish model (Fig. 2I–K). To investigate the impact of *paics* KO on neuromuscular function further, we examined the neuromuscular junction (NMJ) integrity in the larval *paics* F0 KO and control fish. Immunostaining and the analysis of NMJ morphology by NMJ analyser^51^ revealed a significant reduction in the number of presynaptic (SV2a) and postsynaptic (α-BTX) puncta in *paics* F0 KO zebrafish compared with WT zebrafish (Fig. 5C–E). Additionally, we observed a significant reduction in the co-localization of presynaptic (SV2a) puncta with postsynaptic (α-BTX) puncta, in addition to a decrease in the co-localization of postsynaptic (α-BTX) puncta with presynaptic (SV2a) puncta in *paics* F0 KO zebrafish compared with WT zebrafish (Fig. 5C and F). These impaired motor behaviours and NMJ defects in larval *paics* F0 KO zebrafish are consistent with findings that we and others have reported in several other zebrafish models of ALS.^20,88-93^

**Figure 5:**
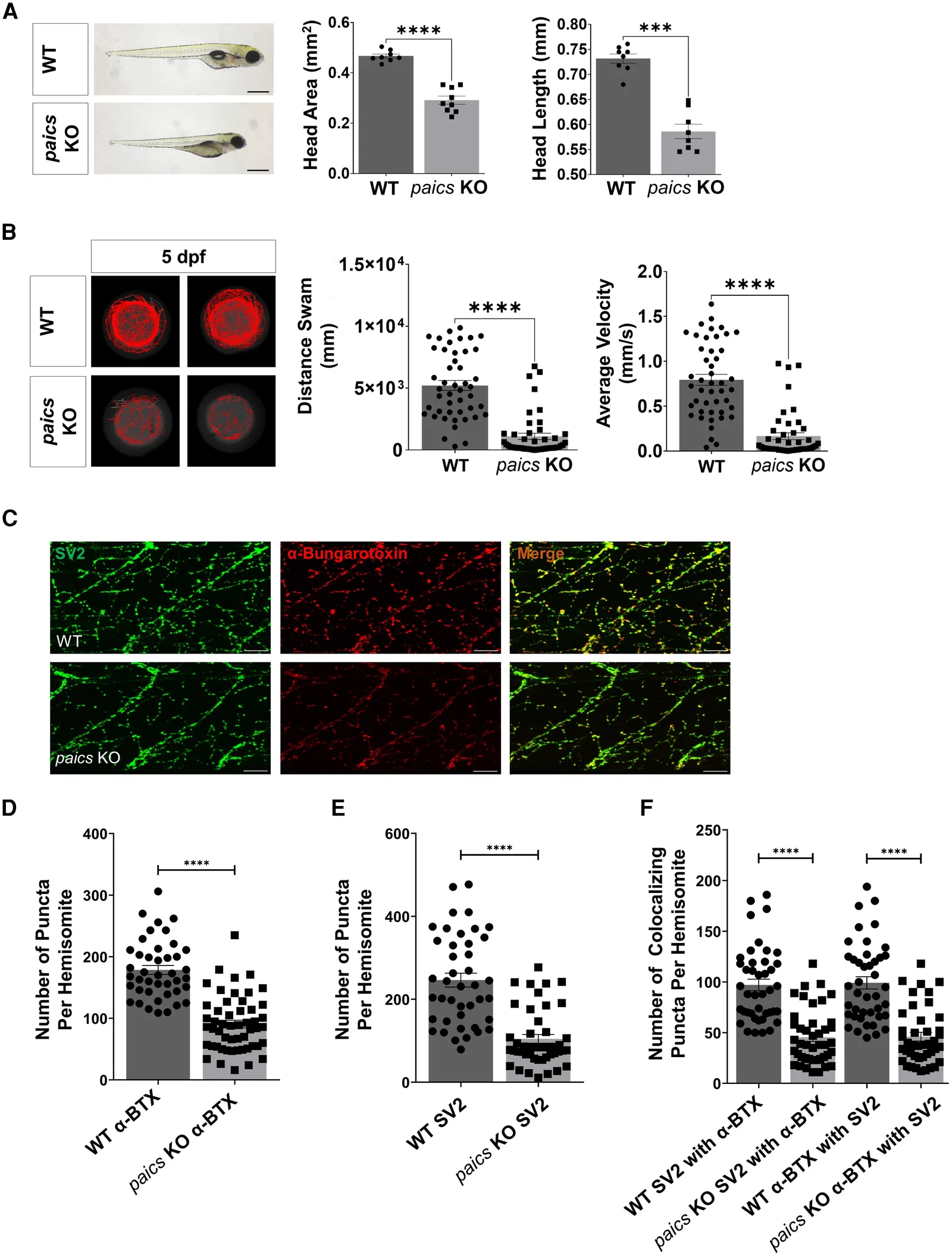
**Morphological, locomotor and neuromuscular junction defects are induced by *paics* knockout in zebrafish.** (**A**) Morphological comparison of willd-type (WT) and *paics* F0 knockout (KO) zebrafish at 5 days postfertilization (dpf). Quantitative analysis includes measurements of head length and head area in both WT (*N* = 8) and *paics* F0 KO (*N* = 8 or 9) zebrafish. Scale bar = 1 mm. (**B**) Representative swimming tracks of WT and *paics* F0 KO zebrafish at 5 dpf. The *paics* F0 KO zebrafish (*n* = 46) displayed a significant decrease in swimming distance and average velocity compared with WT zebrafish (*n* = 46) at 5 dpf. (**C**) Co-immunostaining of zebrafish neuromuscular junctions with presynaptic (SV2a; green) and postsynaptic (α-bungarotoxin; red) markers at 5 dpf. Scale bar = 20 µm. (**D**) Quantitative analysis demonstrated a significant decrease in the number of postsynaptic (α-BTX) puncta in *paics* F0 KO zebrafish compared with WT zebrafish. (**E**) Quantitative analysis demonstrated a significant decrease in the number of presynaptic (SV2a) puncta in *paics* F0 KO zebrafish compared with WT zebrafish. (**F**) Co-localization analysis revealed a significant decrease in the co-localization of presynaptic (SV2a) puncta with postsynaptic (α-BTX) puncta in *paics* F0 KO zebrafish compared with WT zebrafish. Likewise, co-localization analysis of postsynaptic (α-BTX) puncta with presynaptic (SV2a) puncta revealed a marked reduction in co-localizing puncta in *paics* F0 KO zebrafish compared with WT zebrafish. *n* = 42–52. All data are represented as the mean ± standard error of the mean. Statistical significance was calculated by Student’s *t*-test. ns = non-significant; *P < 0.05, **P < 0.01, ***P < 0.001, ****P < 0.0001. α-BTX = α-bungarotoxin; *n* = number of hemisomites.

### Elevated DNA damage results in GABAergic and Purkinje cell loss in *paics* knockout zebrafish cerebellum

We next sought for any alterations in the brain and cerebellar structures in *paics* F0 KO zebrafish. Analysis of GABAergic and glutamatergic neuronal networks in the *paics* F0 KO revealed a significant reduction in the number of GABAergic neurons in the optic tectum and the cerebellum of the *paics* F0 KO zebrafish brain at 5 dpf (Fig. 6A–C), which parallels our observations in the C9-*mir* zebrafish brain. Furthermore, we observed a reduction in the number of glutamatergic neurons only in the optic tectum, but no significant changes were observed in the cerebellum and the hindbrain of the *paics* F0 KO zebrafish brain at 5 dpf (Fig. 6A and D–F). Consistent with our previous observations, we found a significant reduction in the number of pvalb7^+^ PCs in the cerebellum of *paics* F0 KO zebrafish larvae at 5 dpf (Fig. 6G and H), precisely phenocopying our observations of PCs loss in C9-*mir* zebrafish.

**Figure 6:**
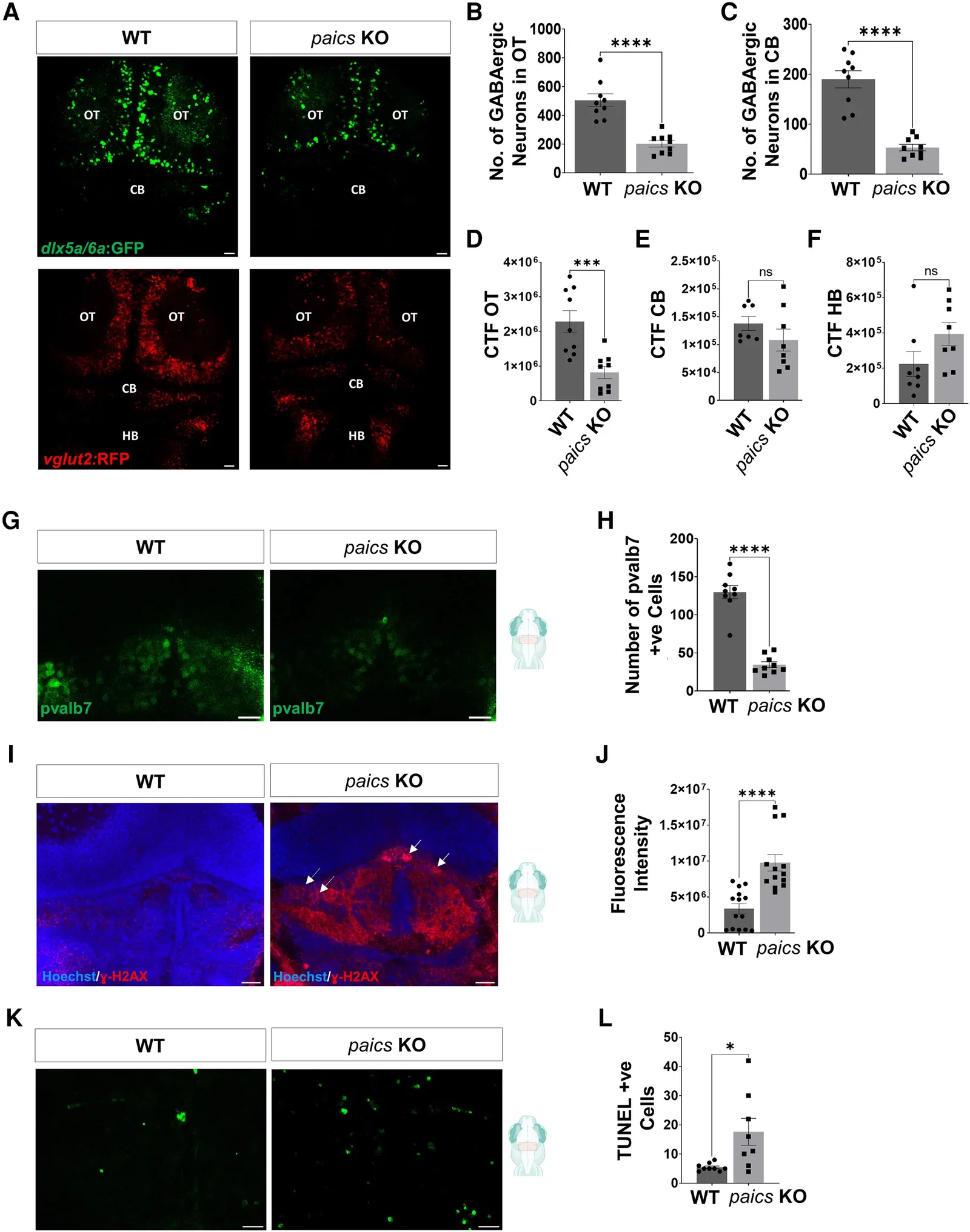
**GABAergic and Purkinje cell loss and increased DNA damage in zebrafish cerebellum after *paics* knockout.** (**A**) Representative images of GABAergic neurons (*dlx5a/6a*:GFP) and glutamatergic neurons (*vglut2*:RFP) in wild-type (WT) versus *paics* F0 knockout (KO) zebrafish brain at 5 days postfertilization (dpf). Scale bar = 20 µm. (**B**) Quantitative analysis demonstrated a significant decrease in the number of GABAergic neurons (*dlx5a/6a*:GFP) in *paics* F0 KO zebrafish compared with WT zebrafish optic tectum at 5 dpf, *n* = 9. (**C**) Quantitative analysis demonstrated a significant decrease in the number of GABAergic neurons (*dlx5a/6a*:GFP) in *paics* F0 KO zebrafish compared with WT zebrafish cerebellum at 5 dpf, *n* = 9. (**D**) Quantitative analysis demonstrated significant decrease in the corrected total fluorescence of glutamatergic neurons (*vglut2*:RFP) in *paics* F0 KO zebrafish compared with WT zebrafish optic tectum at 5 dpf, *n* = 9. (**E**) Quantitative analysis demonstrated no significant changes in the corrected total fluorescence of glutamatergic neurons (*vglut2*:RFP) in *paics* F0 KO zebrafish compared with WT zebrafish cerebellum at 5 dpf, *n* = 7 or 8. (**F**) Quantitative analysis demonstrated no significant changes in the corrected total fluorescence of glutamatergic neurons (*vglut2*:RFP) in *paics* F0 KO zebrafish compared with WT zebrafish hindbrain at 5 dpf, *n* = 8. (**G**) Immunostaining of Purkinje cells (pvalb7^+^) in WT and *paics* F0 KO zebrafish brain at 5 dpf. Scale bar = 10 µm. (**H**) Quantitative analysis demonstrated a significant decrease in the number of Purkinje cells (pvalb7^+^) in *paics* F0 KO zebrafish compared with WT zebrafish cerebellum at 5 dpf, *n* = 9. (**I**) Immunolabelling of γ-H2AX in WT and *paics* KO zebrafish cerebellum at 5 dpf. White arrows represent DNA damage foci, and dashed white arrows represent DNA damage apoptotic rings. Scale bar = 20 µm. (**J**) Quantitative analysis revealed significant upregulation in γ-H2AX signal in *paics* F0 KO zebrafish cerebellum at 5 dpf, *n* = 13 or 14. (**K**) Representation of TUNEL-positive cells in WT and *paics* KO zebrafish cerebellum at 5 dpf. Scale bar = 20 µm. (**L**) Quantitative analysis revealed a significant increase in the number of apoptotic cells in *paics* F0 KO zebrafish versus WT zebrafish cerebellum at 5 dpf, *n* = 8 or 9. All data are represented as the mean ± standard error of the mean. Statistical significance was calculated by Student’s *t*-test. ns = non-significant; *P < 0.05, **P < 0.01, ***P < 0.001, ****P < 0.0001. CB = cerebellum; HB = hindbrain; OT = optic tectum. Figure partially created in BioRender. Patten, K. (2026) https://BioRender.com/ndmvh57.

The human *PAICS* gene has been implicated in regulating DNA damage and repair (DDR).^94,95^ Accumulation of DNA damage has been reported in ALS^96-98^ and other movement disorders, such as Parkinson’s disease^99,100^ and cerebellar ataxia.^101^ To explore this possibility, we investigated whether *paics* loss alone could trigger DNA damage in our KO model. In the *paics* KO zebrafish, we found that the γ-H2AX signal was significantly enhanced in the cerebellum at 5 dpf, supported by an increase in γ-H2AX foci and rings, a marker of double-strand breaks, reflecting increased DNA damage (Fig. 6I and J). At this stage, this corresponded to an enhanced number of TUNEL-positive cells, showing increased apoptosis within the cerebellum of the *paics* KO zebrafish (Fig. 6K and L). These findings suggest that *paics* is a critical mediator of DNA damage that results in cerebellar degeneration.

### Loss of *paics* suppresses key DNA repair pathways in *paics* knockout and C9-*mir* zebrafish

To unravel the molecular underpinnings of the DNA damage in the *paics* KO zebrafish, we carried out qPCR analysis for 18 genes associated with DDR pathways.^102-106^ Transcriptional profiling of these genes revealed a marked upregulation of DNA damage sensors, including *atm*, *atr*, *tp53* and and *rad17*, in *paics* KO zebrafish, indicating an activated DNA damage response (Fig. 7A). However, genes involved in DNA repair processes were significantly downregulated. Specifically, we observed reduced expression of genes critical for base excision repair (*ogg1* and *apex1*), nucleotide excision repair (*ercc1* and *ddb2*) and mismatch repair (*mlh1*, *msh2* and *msh6*) (Fig. 7A). We also found reduced expression of regulatory kinases (*chek1* and *chek2*) that are critical for coordinating DDR signalling, which suggests compromised checkpoint activation (Fig. 7A). Checkpoint kinase dysregulation, as shown by reduced *chek1* and *chek2* expression in the *paics* KO zebrafish, might further exacerbate ALS pathology by impairing cell cycle arrest and apoptosis.

**Figure 7:**
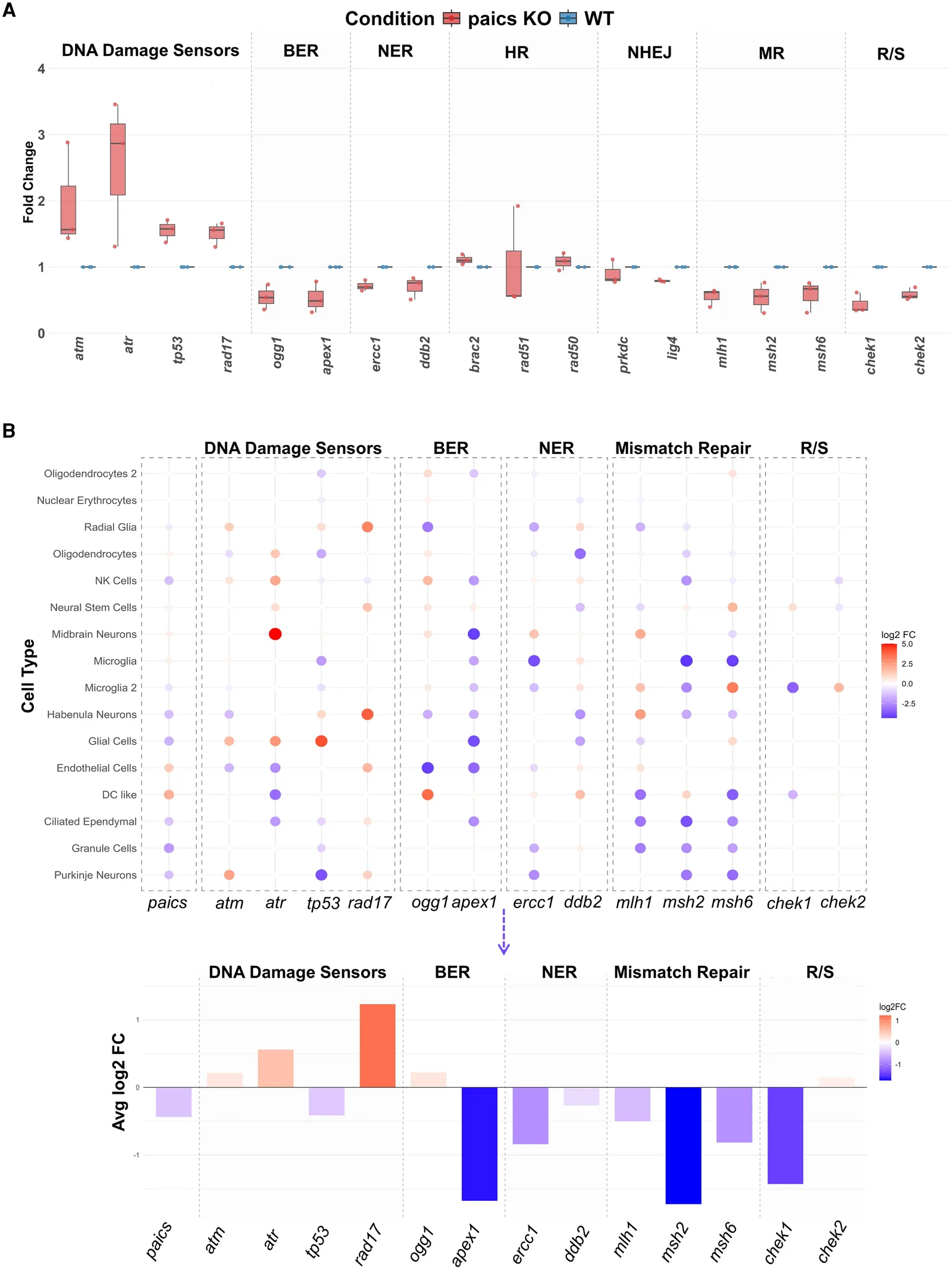
**Suppression of DNA repair pathways in *paics* F0 knockout and C9-*mir* zebrafish.** (**A**) Box plot illustrating the relative mRNA expression levels of DNA damage response and repair (DDR) pathway genes in wild-type (WT) and *paics* F0 knockout (KO) zebrafish. The *x*-axis represents genes categorized by functional pathway [DNA damage sensors, base excision repair (BER), nucleotide excision repair (NER), homologous recombination (HR), non-homologous end joining (NHEJ), mismatch repair (MR) and regulatory/signalling (R/S)], while the *y*-axis denotes the fold change (FC) relative to WT. Data are derived from qPCR analysis (*N* = 3 biological replicates). Each box plot displays the median (centre line), interquartile range (box) and individual data points (dots). Red and blue colours denote *paics* KO and WT conditions, respectively. (**B**) Dot plot and bar graph illustrating cell type-specific and averaged mRNA expression changes of DDR genes in C9-*mir* zebrafish brain. The dot plot (*top*) displays the log_2_FC of gene expression across 16 cell types (*y*-axis), categorized by functional pathway (DNA damage sensors, BER, NER, MR and R/S). Each circle represents the log_2_FC for a specific gene (*x*-axis) in a cell type, with colour intensity indicating magnitude (blue, downregulated; red, upregulated). The bar graph (*bottom*) shows the average log_2_FC across all cell types for each gene. Data were derived from single-cell RNA sequencing analysis. All data are represented as the mean ± standard error of the mean.

To resolve cell type-specific contributions, we analysed the expression of genes involved in DDR across distinct cell types in our single-cell data of C9-*mir* zebrafish brain. The data revealed heterogeneous expression patterns, yet the overall aggregate analysis (average expression across all cell types) aligned with our initial qPCR results in *paics* KO zebrafish. DDR sensors (*atm*, *atr* and *rad17*) showed elevated average expression, although *tp53* was an exception, displaying a slight decrease in the expression (Fig. 7B). For base excision repair pathways, *apex1* expression was markedly reduced, whereas the expression of *ogg1* exhibited a modest but inconsistent upregulation in selected cell types (Fig. 7B). In nucleotide excision repair and mismatch repair pathways, the expression of the genes was predominantly downregulated across cell types, with average expression levels of *ercc1*, *ddb2*, *mlh1*, *msh2* and and *msh6* showing substantial reductions (Fig. 7B). Regulatory kinases exhibited divergent trends, whereby *chek1* expression was greatly decreased, whereas *chek2* showed a subtle increase in average expression (Fig. 7B).

This convergence of DDR defects across C9-*mir* and *paics* KO zebrafish further underscores *paics* as a critical mediator of DNA damage pathology in C9orf72-associated ALS. The upregulation of DDR sensors and the persistent presence of unresolved DNA damage, as observed through γ-H2AX accumulation, aligns with findings from ALS patient-derived tissues.^96,97,107,108^ Additionally, impaired base excision repair (BER) genes (*ogg1* and *apex1*) contribute to oxidative DNA damage, a hallmark of ALS.^98^  ^,109,110^ Reduced expression of mismatch repair (MMR) genes (*mlh1*, *msh2* and *msh6*) compromise genomic integrity as slippage in repetitive sequences, mirroring microsatellite instability, another characteristic hallmark of C9orf72-associated ALS.^111,112^ Critically, the loss of *paics* phenocopies C9orf72-associated ALS at the genomic level, positioning purine metabolism as a linchpin connecting nucleotide pool imbalance, DDR failure and ALS progression.

### *paics* overexpression rescues Purkinje and granule cell loss and DNA damage response in C9-*mir* zebrafish

Given the critical role of *paics* in maintaining cerebellar neuronal integrity and DNA repair, we examined whether restoring the expression of *paics* could rescue the cerebellar deficits observed in C9-*mir* zebrafish. We cloned the WT zebrafish *paics* coding sequence into the pCS2+ plasmid and, from that, we generated the capped WT *paics* mRNA (*paics*^WT^) using *in vitro* transcription and injected it into C9-*mir* zebrafish embryos at the one-cell stage (Supplementary Fig. 6A). Immunostaining for pvalb7 and neurod1 revealed a significant reduction in the number of PCs and GCs in the cerebellum of C9-*mir* larvae compared with controls at 5 dpf, which was consistent with our previous observations (Fig. 8A–C). Remarkably, overexpression of *paics*^WT^ mRNA into C9-*mir* zebrafish resulted in a robust rescue of the number of PCs and GCs to levels comparable to control larvae (Fig. 8A–C).

**Figure 8:**
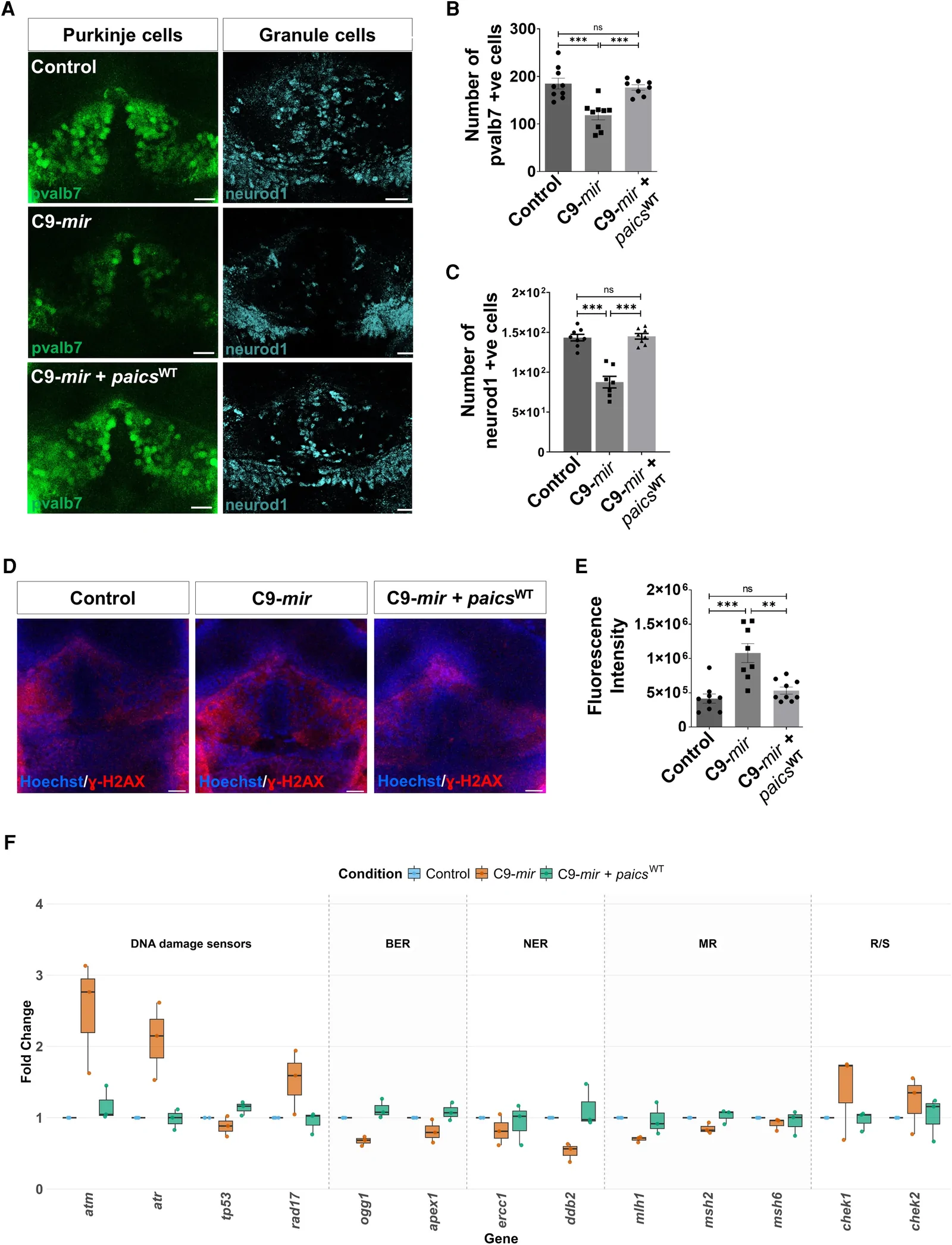
**Injection of *paics* mRNA rescues Purkinje and granule cell loss and DNA damage response in C9-*mir* zebrafish.** (**A**) Immunostaining of Purkinje cells (pvalb7^+^) and granule cells (neurod1^+^) in control, C9*-mir* and C9-*mir + paics*^WT^ zebrafish cerebellum at 5 days postfertilization (dpf). Scale bar = 20 µm. (**B**) Quantitative analysis demonstrated a significant decrease in the number of Purkinje cells (pvalb7^+^) in C9-*mir* zebrafish compared with control zebrafish cerebellum at 5 dpf. Injection of *paics* mRNA into C9-*mir* zebrafish significantly restored Purkinje cell numbers, reaching levels comparable to those of control larvae, *n* = 8 or 9. (**C**) Quantitative analysis demonstrated a significant decrease in the number of granule cells (neurod1^+^) in C9-*mir* zebrafish compared with control zebrafish cerebellum at 5 dpf. Injection of *paics* mRNA into C9-*mir* zebrafish significantly restored granule cell numbers, reaching levels comparable to those of control larvae, *n* = 7 or 8. (**D**) Immunolabelling of γ-H2AX to detect DNA damage in control, C9*-mir* and C9-*mir + paics*^WT^ zebrafish cerebellum at 5 dpf. Scale bar = 20 µm. (**E**) Quantitative analysis demonstrated a significant upregulation in γ-H2AX signal in C9-*mir* zebrafish compared with control zebrafish cerebellum at 5 dpf. Injection of *paics* mRNA into C9-*mir* zebrafish significantly mitigated the DNA damage, reaching levels comparable to those of control larvae, *n* = 8 or 9. (**F**) Box plot illustrating the relative mRNA expression levels of DNA damage response genes in control, C9-*mir* and C9-*mir + paics*^WT^ zebrafish. The *x*-axis represents genes categorized by functional pathway [DNA damage sensors, base excision repair (BER), nucleotide excision repair (NER), mismatch repair (MR) and regulatory/signalling (R/S)], while the *y*-axis denotes the fold change relative to controls. Data are derived from qPCR analysis (*N* = 3 biological replicates). Each box plot displays the median (centre line), interquartile range (box) and individual data points (dots). Blue, orange and teal denote control, C9-*mir* and C9-*mir + paics*^WT^ conditions, respectively. All data are represented as the mean ± standard error of the mean. Statistical significance was calculated by one-way ANOVA followed by Dunnett’s T3 multiple comparisons test. ns = non-significant; *P < 0.05, **P < 0.01, ***P < 0.001, ****P < 0.0001. WT = wild-type.

Given that the downregulation of *paics* was associated with increased DNA damage in *paics* KO zebrafish, we next assessed whether overexpression of *paics*^WT^ mRNA could also mitigate DNA damage in C9-*mir* larvae. Immunostaining for γ-H2AX, a marker of DNA double-strand breaks, revealed extensive DNA damage in the cerebellum of C9-*mir* zebrafish at 5 dpf (Fig. 8D and E). However, *paics*^WT^ mRNA-injected C9-*mir* larvae exhibited a significant reduction in the γ-H2AX signal, with levels comparable to control fish (Fig. 8D and E).

Given that loss of *paics* in both *paics* KO and C9-*mir* zebrafish was associated with activation of DNA damage sensors and suppression of multiple DNA repair pathways, we next asked whether the restoration of *paics* expression via mRNA injection could reverse transcriptional dysregulation of DDR genes in the C9-*mir* zebrafish. We therefore measured the expression of the same panel of 13 DDR genes involved in DNA damage sensing and repair. In C9-*mir* larvae, we observed the characteristic pattern of marked upregulation of DDR sensors (*atm*, *atr* and *rad17*) together with reduced expression of base excision repair (*ogg1* and *apex1*), nucleotide excision repair (*ercc1* and *ddb2*) and mismatch repair (*mlh1* and *msh2*) at 6 dpf (Fig. 8F). Strikingly, we observed the upregulation of checkpoint regulators (*chek1* and *chek2*) in the C9-*mir* zebrafish at 6 dpf (Fig. 8F). The injection of *paics*^WT^ mRNA to restore *paics* expression in C9-*mir* zebrafish largely normalized the DDR profile by reducing the elevated DNA damage sensor transcripts and restoring the expression of DNA repair and checkpoint genes towards control levels (Fig. 8F).

Together, these findings demonstrate that restoration of *paics* expression is sufficient to rescue both PC and GC loss and DDR pathways in C9-*mir* zebrafish, positioning *paics* as a critical mediator of C9orf72-associated cerebellar pathology in ALS.

### *paics* overexpression ameliorates motor deficits in C9-*mir* zebrafish larvae

Having established that *paics* overexpression can rescue cerebellar cellular populations and DDR pathways in C9-*mir* zebrafish, we next sought to test whether motor function can also be rescued. Consistent with our previous observations, C9-*mir* zebrafish exhibited notable motor deficits at 6 dpf, with a significant reduction in the distance travelled and average velocity of C9-*mir* zebrafish compared with control zebrafish at 6 dpf (Supplementary Fig. 6B and C). Remarkably, injection of *paics*^WT^ mRNA in C9-*mir* zebrafish showed a significant rescue of swimming distance and average velocity, reaching values comparable to those of control larvae (Supplementary Fig. 6B and C). These data indicate that *paics* overexpression not only corrects cerebellar degeneration and DNA damage phenotypes but also confers a robust functional rescue at the level of motor behaviour.

## Discussion

In this study, we demonstrate that the C9orf72 LOF in zebrafish initiates a cascade of neurodegenerative events that extends beyond motor neuron pathology. Our study provides new insights into the early cerebellar pathology resulting from C9orf72 LOF, revealing key mechanistic links between *paics*, a gene involved in *de novo* purine biosynthesis and DDR failure.

We show that C9orf72 LOF in zebrafish leads to global brain atrophy, selective reduction in GABAergic neurons and loss of PCs and GCs prior to motor dysfunction. These findings align with clinical neuroimaging studies of cerebellar atrophy and cerebro-cerebellar connectivity changes in C9orf72-ALS patients.^23,24,35^ Notably, our work extends earlier findings of cerebellar RNA foci and dipeptide repeat protein deposition in ALS^29^ to demonstrate that *C9orf72* haploinsufficiency is sufficient to cause cerebellar vulnerability independently.

A crucial mechanistic insight from our study is the identification of disrupted purine metabolism as a factor contributing to cerebellar pathology in ALS. Through the single-cell transcriptomics and GSEA, we observed a disruption in the purine-containing compound biosynthetic process in the PCs of C9-*mir* zebrafish, characterized by downregulation of the *paics* gene, which encodes an essential enzyme in the *de novo* purine biosynthesis pathway. Crucially, this transcriptional reduction translates to the proteomic level, in that we observed a significant downregulation of Paics protein in the C9-*mir* zebrafish. Interestingly, a recent KEGG pathway analysis in a separate study^94^ identified ALS as one of the top-enriched pathways among 4039 genes co-expressed with *PAICS* in non-small cell lung cancer. Our *paics* KO study, which recapitulated the cerebellar pathology and motor defects of the C9-*mir* zebrafish, also highlights the link between metabolic imbalance and the extensive dysregulation of DDR. The upregulation of DNA damage sensors and the downregulation of key DNA repair genes, especially the genes involved in the mismatch repair pathway, suggest that disrupted purine homeostasis can exacerbate genomic instability, thus propagating neurodegeneration in ALS.

Importantly, the translational relevance of our findings is supported by the convergence of molecular and cellular pathology in human ALS patient tissues. We observed a significant downregulation of PAICS protein in the post-mortem cerebellar sections from *C9orf72*-HRE carriers compared with controls. Crucially, this molecular defect was accompanied by a marked reduction in the GABA-AR α1, which is a marker for GCs. This demonstrates that the loss of cerebellar GCs we identified in the C9*-mir* zebrafish is a clinically conserved feature with human ALS cerebellar pathology. We also observed reduced PAICS expression and GC signal in a sporadic ALS case, suggesting that this cerebellar pathology might extend beyond C9orf72-linked ALS.

Given that motor neuron loss is a primary hallmark of ALS, and our observation of global downregulation of Paics protein in C9-*mir* zebrafish, we next investigated whether this purine biosynthesis defect extends to motor neurons. We found that *PAICS* is significantly downregulated at the transcriptomic level in iPSC-derived motor neurons from both sporadic and *C9orf72* ALS patients, with further proteomic analysis confirming reduced PAICS protein levels in *C9orf72* iPSC-derived motor neurons. Collectively, our data indicate that PAICS deficiency is a broad, systemic consequence of C9orf72 LOF rather than a defect restricted to a single cell type. To validate the role of *paics* functionally, we restored the expression of *paics* in C9-*mir* zebrafish via mRNA injection. Remarkably, *paics* overexpression rescued the PC and GC loss and significantly reduced DNA damage and restored DDR pathways in the C9-*mir* zebrafish, to levels comparable to controls. Moreover, the rescue of cerebellar phenotypes resulted in a robust functional rescue of motor behaviour in the C9-*mir* zebrafish. These rescue experiments directly demonstrate that paics downregulation is a key driver of cerebellar neuronal vulnerability and DNA damage downstream of *c9orf72* LOF.

Our data propose a model wherein C9orf72 LOF disrupts purine biosynthesis via *paics* downregulation, leading to the depletion of nucleotides required for DNA repair. This creates a self-perpetuating cycle of unresolved DNA damage (evidenced by γ-H2AX accumulation), repair pathway suppression and neurodegeneration. These defects mirror genomic instability in ALS patients and suggest that *C9orf72* haploinsufficiency converges on DDR failure. Our results make the case for a broader model of ALS pathogenesis that integrates both motor and non-motor systems and combines metabolic defects with genomic instability. Furthermore, this metabolic restriction offers a plausible explanation for the persistence of neurodegeneration in our model, despite the innate regenerative capacity of the zebrafish CNS. Successful regeneration requires rapid proliferation of progenitor cells, a process heavily dependent on available nucleotide pools for DNA replication. We postulate that Paics downregulation creates a metabolic bottleneck, wherein the depletion of purine substrates limits the clonal expansion required for tissue repair. Additionally, the observed upregulation of DNA damage checkpoints (*atm* and *atr*) is likely to trigger cell cycle arrest, effectively locking progenitor cells in a non-proliferative state and preventing compensatory regeneration.

Given that Paics is a critical enzyme in the *de novo* purine biosynthesis pathway, its downregulation leads to the accumulation of specific metabolites, such as formylglycineamide riboside, succinylaminoimidazolecarboxamide riboside and aminoimidazolecarboxamide riboside, in blood, urine and CSF.^86,113^ Furthermore, *PAICS* deficiency has been associated with neurodevelopmental and neuromuscular abnormalities, similar to those seen in ALS.^86^ Therefore, the biochemical analysis and measurement of these metabolites in blood or urine could serve as potential biomarkers for ALS diagnosis and progression.

Despite these insights offered by our study, the precise molecular mechanisms that lead to the downregulation of PAICS in the *C9orf72* LOF need to be elucidated. Our *in silico* modelling with AlphaFold 3 argues against a direct physical interaction between C9orf72 and PAICS (Supplementary Fig. 3B), suggesting an indirect regulation of PAICS. Mechanistically, C9orf72 is required for proper mechanistic target of rapamycin complex 1 (mTORC1) activation at the lysosome through its binding to Rag GTPases.^114^ In addition to that, the loss of C9orf72 function has been shown to decrease mTORC1 signalling and increase transcription factor EB activity, indicating a metabolic switch away from anabolic programmes.^115^ Given that mTORC1 signalling is a master driver of *de novo* purine synthesis^116-118^ and promotes the spatial assembly of the ‘purinosome’,^119^ which is a multi-enzyme complex required for efficient purine production, its suppression in *C9orf72*-LOF conditions might lead to diminished PAICS expression and impaired complex assembly. Furthermore, the reduction of PAICS in sporadic ALS iPSC-derived motor neurons and post-mortem cerebellum samples suggests that this defect extends beyond C9orf72-linked ALS. The downregulation of PAICS might represent a convergent consequence of TDP-43 pathology, a hallmark shared by nearly 97% of ALS cases.^120^ The nuclear depletion of TDP-43 has been shown to destabilize the mRNA of Raptor, a key component of the mTORC1 complex,^121^ thereby directly impairing mTORC1 signalling. Given that mTORC1 is the driver of *de novo* purine biosynthesis,^116-118^ its suppression via TDP-43 pathology might lead to the repression of PAICS expression. Thus, familial and sporadic ALS appear to converge on the same purine metabolic defect. However, further research is warranted to validate these upstream signalling cascades definitively and map the mechanistic link between *C9orf72* haploinsufficiency and PAICS downregulation precisely. Moreover, the mechanisms linking the loss of PAICS to DDR failure and neurodegeneration require further work. Studies exploring how purine metabolism dysfunction impairs DNA repair enzymes or alters synaptic function in PCs and GCs are yet to be performed, which might provide us with key insights to target specific pathways via small molecules or gene therapy to stop or even reverse the progression of ALS.

## Conclusion

In conclusion, our study reveals a new mechanistic axis in the pathogenesis of ALS by linking the *C9orf72* haploinsufficiency to cerebellar neurodegeneration via purine biosynthesis disruption and DDR failure. The identification of PAICS as a conserved mediator in the zebrafish and human ALS system highlights its potential as a therapeutic target and a biomarker. These findings not only enhance our understanding of ALS as a multisystem disorder but also open new avenues for therapeutic intervention aimed at restoring metabolic and genomic homeostasis.

## Supplementary Material

awag092_Supplementary_Data

## Data Availability

Cell type markers for each cell type cluster in the single-cell RNA data, DEGs for PCs and GCs and GSEA data have been deposited in the Supplementary material. Additional data are available upon request.
