# Supplementary material for: PAICS mediates DNA damage and cerebellar neuronal loss in C9orf72 amyotrophic lateral sclerosis
Source: Brain. 2026 Mar 11;149(10):3475–94. doi: 10.1093/brain/awag092 (PMC13634648; doi:10.1093/brain/awag092)
Supplement: Supplementary file 1 [file awag092_supplementary_data.zip › brain-2025-01736-File010.pdf]

# PAICS mediates DNA damage and cerebellar neuronal loss in C9orf72 amyotrophic lateral sclerosis

Jaskaran Singh,[1] Léa Lescouzères,[1] Charlotte Zaouter,[1] Mathilde Chaineau,[2] Ghazal Haghi,[2] Thomas M. Durcan[2] and Shunmoogum A. Patten[1,3]

**Author affiliations:**

1 Institut National de la Recherche Scientifique (INRS), Centre Armand Frappier Santé Biotechnologie, Laval, QC H7V 1B7, Canada

2 The Neuro's Early Drug Discovery Unit (EDDU) McGill University, Montreal, QC, Canada

3 Département de Neurosciences, Université de Montréal, Montréal, QC H3C 3J7, Canada

Correspondence to: Dr Shunmoogum (Kessen) Patten

Institut National de la Recherche Scientifique (INRS)

531, Boul. des Prairies, Laval, Quebec, Canada, H7V 1B7

E-mail: kessen.patten@inrs.ca

# Supplementary materials and methods

## Zebrafish

### *In vitro* transcription and rescue experiments

The full length coding sequence of the zebrafish *paics* gene was amplified from WT zebrafish cDNA. The pCS2+ vector was linearized using SapI and purified. Gibson assembly was performed using the Gibson assembly cloning kit (New England Biolabs, E5510S) according to the manufacturer's instructions. Briefly, the linearized pCS2+ vector and the *paics* PCR product, designed with overlapping sequences, were incubated at 50°C for 30 minutes in the Gibson assembly master mix. Following assembly, competent *E. coli* DH5α cells were transformed with the Gibson reaction mix and plated on LB agar plates containing ampicillin (100 µg/mL). Single colonies were picked and expanded in LB broth with ampicillin. The plasmid DNA was isolated using the QIAprep Spin Miniprep Kit (Qiagen, 27104) according to the manufacturer's instructions. Correct insertion of the *paics* sequence was confirmed by sanger sequencing.

For *in vitro* transcription (IVT), the plasmid DNA from a confirmed positive clone was linearized with DraIII, purified, and used as a template for capped mRNA synthesis using the mMESSAGE mMACHINE™ SP6 Transcription Kit (Thermo Fisher Scientific, AM1340), following the manufacturer's protocol. Capped mRNA was synthesized to ensure transcript stability and efficient translation in zebrafish embryos.

The mRNA was diluted to a final concentration of 20 ng/µl in RNsase free water. Approximately 1 nL of *paics* mRNA (20 ng/µl) was microinjected into one-cell stage C9-*mir* zebrafish embryos.

### Morphology assessment

WT and *paics* KO larvae were anesthetized using tricaine methanesulfonate (MS222). Dorsal and lateral images of the whole body were acquired under a Leica S6E stereomicroscope. Zebrafish larvae were assessed for head length and head area. The measurements were done using the software, ImageJ.

## Motor behaviour assay

For the zebrafish motor behaviour assessment, individual zebrafish larvae were placed in a 96-well plate containing 200 µl of E3 medium. The DanioVision observation chamber with Basler GenIcam was used for monitoring their activities. All larvae were allowed to acclimate for 30 minutes in the chamber before the recording. Motor behaviour parameters such as total swimming distance and average velocity were quantified using EthoVision XT software.

## Hematoxylin and eosin staining

For Hematoxylin and eosin staining, 10-µm cryosectioned zebrafish brain sections were post-fixed in 10% formalin for 5 minutes and washed with tap water. The sections were then stained with hematoxylin for 4 minutes, followed by a wash with alcohol-hydrochloric acid solution, and washed again with tap water. Subsequently, the sections were immersed in a saturated lithium carbonate solution for 10 seconds and washed with tap water. Finally, sections were counterstained with eosin Y for 2 min and mounted under coverslips in the mounting medium.

## Neural network analysis

To image and quantify GABAergic neurons (*dlx5a/6a*:GFP) and glutamatergic neurons (*vglut2*:RFP) in 5 dpf zebrafish larvae of Tg (*dlx5a/6a*:GFP,control); Tg (*vglut2*:RFP, control), Tg (*dlx5a/6a*:GFP,C9-*mir*); Tg (*vglut2*:RFP, C9-*mir*) and (*dlx5a/6a*:GFP,*paics* KO); Tg (*vglut2*:RFP, *paics* KO) were fixed in 4% PFA for 20 mins. Zebrafish larvae were mounted in 0.5% low-melting point agarose (Invitrogen; 16520-100). Z-stack images were taken using a Zeiss LSM780 confocal microscope (Carl Zeiss, Germany). GABAergic neurons *(dlx5a/6a*:GFP) were counted in the brain regions of control and C9-*mir* zebrafish manually and blindly using the Cell Counter plug-in for Fiji/Image J (NIH) Imaging software. Corrected total fluorescence was measured for the analysis of glutamatergic neurons (*vglut2*:RFP) in a brain region-wise manner as per the described protocol by the open lab book [1].

## TUNEL assay

Zebrafish larvae were fixed overnight in 4% PFA at 4°C. The next day, three 5 min washes of 0.1% Tween-20/PBS (PBS-Tween) were given. Proteinase K (Prot-K) treatment was performed at 30°C for 20 minutes using concentration of 37.5 µg/ml for 5 dpf zebrafish larvae. After three 3-

min PBS-Tween washes, larvae were refixed in 4% PFA for 20 minutes, followed by two 5-min PBS-Tween washes. Permeabilization was achieved by incubating in 1% Triton X-100/PBS for three 20-min intervals on a rotator, followed by three quick PBS washes. Larvae were then incubated in TUNEL reaction mixture (1:9 ratio of enzyme to labelling solution; 50 µL per tube containing 5-8 fish) for 1 hour at 37°C followed by two final 5 min PBS washes. The larvae were then mounted in 0.5% low-melting point agarose (Invitrogen; 16520-100) and imaged using a Zeiss LSM 780 confocal microscope (Carl Zeiss, Germany).

## Real time-quantitative PCR

RNA was isolated from ~ 30 embryos using TriReagent® (Sigma) according to manufacturer's protocol. 1 µg of RNA was used for cDNA synthesis by SuperScript®Vilo™ kit (Invitrogen). RT–qPCR was run with SYBR Green Master Mix (Bioline) using the LightCycler® 96 (Roche). Tests were run in triplicates of at least three independent experiments.

## Western Blotting

The protein lysates were prepared from pools of 20 zebrafish larvae at 5 dpf. The larvae were then homogenized in 150 µL of lysis buffer (150 mM NaCl, 50 mM Tris-HCl pH 7.5, 1% Triton X-100, 0.1% SDS, 1% sodium deoxycholate) supplemented with a protease inhibitor (1:10; Sigma-Aldrich). Then the samples were centrifuged at 10,000 rpm for 10 min at 4 °C, and the supernatant was collected. Protein concentration was quantified using the Bradford assay (BioRad). For immunoblotting, 40 µg of protein per sample was resolved on a 7.5% SDS-polyacrylamide gel (BioRad) and electrotransferred onto polyvinylidene difluoride (PVDF) membranes (BioRad). Then the membranes were blocked with either 5% non-fat milk in phosphate-buffered saline (PBS) and incubated overnight with anti-PAICS primary antibody (1:1000; Proteintech, 12967-1-AP). Following incubation with a horseradish peroxidase (HRP)-conjugated goat anti-rabbit secondary antibody, the protein bands were visualized using enhanced chemiluminescence (ECL) and imaged on a ChemiDoc system (BioRad). Densitometric quantification was performed using Image Lab software (BioRad), with data normalized to total protein loading [2].

## Brain Dissection and tissue dissociation

WT and C9-*mir* adult zebrafish (2 years-old) were euthanized by immersion in an excess concentration of Tricaine (Sigma). Brain were dissected and transferred to a 24-well plate

containing NbActiv1 medium (Neurobasal™ supplemented with B27™ and Glutamax™; BrainBits, #NC0319856, Fisher) on ice. Tissues were briefly washed twice with FACSmax solution (#T200100, Genlantis) to remove debris. For enzymatic dissociation, samples were incubated in FACSmax for 20 minutes on ice. Mechanical dissociation was performed by gentle trituration with a 1 mL pipette tip, avoiding excessive shear stress to maintain cell integrity. Cell suspensions were centrifuged at 500g for 5 minutes at 4°C. Pellets were washed twice with NbActiv1. Cell suspensions were passed through 40-μm cell strainer (CORNING) into a 50 mL tube by gently pipetting, and remaining tissue fragments were crushed against the strainer using the flat end of a syringe. The filtered cell suspension was centrifuged again for 5 minutes at 4°C, and the final cell pellet was resuspended in NbActiv1 for downstream applications.

## RNA Extraction and Reverse Transcription

Single-cell RNA was captured using poly[T]-primed beads to selectively isolate polyadenylated mRNA molecules. Extracted RNA was reverse-transcribed to cDNA using reverse transcriptase and primers containing adaptor sequences compatible with next-generation sequencing (NGS) platforms, unique molecular identifiers (UMIs), and cellular barcodes. This step ensured that each mRNA molecule and its cellular origin were uniquely tagged.

## cDNA Amplification and Library Preparation

Amplified cDNA was obtained by PCR. Libraries were prepared with nucleotide barcode tagging to ensure accurate identification of individual cells. Amplified cDNA libraries from all cells were pooled and sequenced an Illumina NovaSeq 6000 at the McGill Genome Centre Platform.

## Quantitative PCR

Total RNA was isolated from differentiated MNs using TriReagent® (Sigma) according to manufacturer’s protocol. 1μg of purified RNA was used for cDNA synthesis using the SuperScript®Vilo™ kit (Invitrogen; Cat#11754050). RT-qPCR reactions were run in triplicates with SYBR Green Master Mix (Biorad) using the LightCycler® 96 (Roche). GAPDH was used as the reference gene for normalization.

## Single-cell RNA data analysis

The bioinformatics analysis was conducted using R (version 4.3.1) with a focus on Seurat version 5 [3]. Quality control measures involved cell scoring through PercentageFeatureSet for mitochondrial genes (^mt-). Cells were discarded if they exhibited low sequencing depth (features ≤ 200) or had elevated mitochondrial content (≥14-18%). To eliminate potential multiplets, cells with excessive sequencing depth were filtered out (features ≥ 2500; counts ≥10000). Features that were absent in at least three cells were also removed from the analysis. The Seurat RNA assay data underwent log-normalization using the default parameters of NormalizeData and was scaled across all the remaining genes. After normalization and scaling, we further applied principal component analysis (PCA) to reduce data dimensionality, thus identifying the major sources of variation. The Elbow Plot method was used for choosing the number of principal components for downstream analysis, where principal components are ranked by the percentage of variance explained, enabling the selection of significant principal components through the identification of an 'elbow' point in the plot. Additionally, we also used the JackStraw method, which computes the robustness of the PCA by contrasting the composition of the principal components to the composition of principal components computed from permuted data, for statistical assessment of the significance of each principal component.

Identification and exclusion of doublets, artificially merged cells that may confound single-cell RNA sequencing analyses, were performed using the DoubletFinder package [4]. We first determined the optimal pK value, a critical parameter defining the size of the neighbourhood for doublet detection. This was obtained by performing a parameter sweep using the paramSweep function, followed by summarization with summarizeSweep. We thereafter identified the pK value that maximizes the mean-variance-normalized bimodality coefficient (BCmvn), since this value optimizes DoubletFinder's accuracy. Next, we estimated the proportion of homotypic doublets-doublets formed by cells of the same type, using the modelHomotypic function, which leverages existing cell annotations to model this proportion. Based on this estimate, we updated the expected number of doublets due to the possible presence of homotypic doublets. Using these parameters of pN and pK, along with the newly estimated count of doublets, we used the doubletFinder function to classify cells as a doublet or singlet. These steps are crucial for ensuring the accuracy of downstream analyses by identifying and removing potential doublets.

Cluster annotation and identification involved multiple steps to ensure the accurate identification of cell types. The conserved markers were identified for each cluster using the

FindConservedMarkers function. We utilised marker-based annotation strategy to annotate cell clusters based on marker gene expression. Differentially expressed genes (DEGs) were identified to explore transcriptomic differences between conditions (C9-*mir* vs. WT) for each cell type. The FindMarkers function was used to compute DEGs for each cluster across conditions.

### Gene set enrichment analysis of PCs and GCs

PCs cluster was obtained by subsetting the cells expressing *ca8* as a marker gene using the subset function from the main clusters and differential expression was performed using the FindMarkers function to compute DEGs in PCs. GC cluster was already present in the main cell clusters. We used R (version 4.3.1), gene pattern and fgsea package for the GSEA [5]. We imported gene sets from a GMT file and concurrently, a CSV file containing DEGs (filtered for threshold of >20% for PCs and >40 % for GCs cells expressing the gene ), was transformed into a named vector (geneList) of average log2 fold changes, sorted in descending order. Utilizing the gene pattern platform and fgsea package in R, we performed GSEA with parameters set to a minimum gene set size of 15 and a maximum of 500. The results were filtered to retain pathways with a p-value below 0.05 and an FDR value <0.25 for GCs and <0.40 for PCs. To visualize, we employed the cnetplot function from the enrichplot package and visualized the top enriched pathways and their corresponding genes in a network plot. The colour gradient displays the direction and magnitude of fold change: red represents upregulated and blue represents downregulated.

## iPSC-derived motor neurons

### Immunofluorescence staining

For immunofluorescence staining, the cells were plated on 12-mm circular coverslips, fixed in 4% formaldehyde for 20 min at room temperature and washed three times with PBS. Cells were then permeabilized with 0.2% Triton X-100 in PBS for 10 min and blocked for 1 hour at room temperature in blocking solution (5% normal donkey serum (NDS, Millipore; Cat#S30-100), 1% bovine serum albumin (BSA, Multicell; Cat#800–095-CG) and 0.05% Triton X-100 in PBS). Cells were incubated with primary antibodies in blocking buffer overnight at 4 °C. After three washes in PBS, cells were incubated with appropriate secondary antibodies for 2 hours at room

temperature, followed by staining with Hoechst DNA dye (Life Technologies; Cat#H3570) for 10 min. Fluorescence images were captured using a confocal laser scanning microscope, model LSM700 (Carl Zeiss) and automated Evos FL-Auto2 imaging system (ThermoFisher Scientific). iPSC-derived MNs visualized by immunocytochemistry with the combined HB9 and Islet1 MN markers (HB9/ISL1), and the neuronal marker neurofilament heavy chain (NF-H) after 21 days of final differentiation (Supplementary Fig. 1A). The following primary antibodies were used: anti-PAICS (Proteintech, Cat#12967-1-AP), anti-Neurofilament-H (Abcam; Cat#ab4680), anti-Hb9 (DSHB; Cat#81.5C10) and Islet-1(DSHB; Cat#40.2D6).

### Western blot

Proteins were collected from differentiated MNs. For Western blotting, 20 µg of protein per sample in Laemmli buffer was resolved on 7.5% or 10% SDS-PAGE gels and transferred to PVDF or nitrocellulose membranes using the Trans-Blot Turbo Transfer System (Bio-Rad). The membranes were blocked for 1 hour with 5% non-fat milk solution in 1× phosphate buffered saline (PBS). Primary PAICS antibody (1:1000; Proteintech, 12967-1-AP) was then applied in blocking solution and incubated overnight at 4°C. After three washes with TBS-Tween (0.1%), membranes were incubated for 2 hours at room temperature with horseradish peroxidase (HRP)-conjugated goat anti-rabbit secondary antibody. Bands were visualized using Clarity Western ECL Substrate or Clarity Max Western ECL Substrate (Bio-Rad; Cat#170–5061, Cat#1705062) and imaged using the ChemiDoc MP Imaging System (Bio-Rad).

## Human postmortem tissues

### Immunostaining of post-mortem cerebellum sections

Paraffin-embedded cerebellar sections were deparaffinized in xylene (5 min × 2) and rehydrated through graded ethanol (100%, 95%, 75%, 50%; 5 min each) followed by two 3-min washes with sterile $H_2O$. Antigen retrieval was performed using Tris-EDTA buffer (10 mM Tris, 1 mM EDTA, pH 9.0) via heat-induced epitope retrieval (HIER). The slides were submerged in preheated buffer (95°C) for 20 min and then cooled to room temperature (RT) for 20 min, and rinsed in PBS. Sections were permeabilized with 0.3% Triton X-100 in PBS (10 min, RT) in a humidified chamber and then blocked with 5% NGS + 2% BSA + 0.3% Triton X-100 in PBS (1 hr, RT). Then,

primary antibodies: Anti-PAICS (1:100, Proteintech:12967-1-AP), Anti-Calbindin (1:200, Proteintech: 66394-1-IG) and Anti- GABA-AR α1 (1:200, allomone labs: AGA-001) were added to the blocking solution and the sections were incubated in it overnight at 4°C. The next day, following three 15 min PBST (PBS + 0.1% Tween 20) washes, the sections were incubated with species-specific secondary antibodies diluted in blocking solution coupled to Alexa Fluor 488 or 568 (1:500, Invitrogen) overnight at 4°C. The following day, sections were washed three times in PBST (15 min each, dark) and counterstained with Hoechst. Sections were mounted in fluoromount-G (Invitrogen 00–4958-02) and imaged using a Zeiss LSM 980 confocal microscope (Carl Zeiss, Germany).

# Supplementary Figures

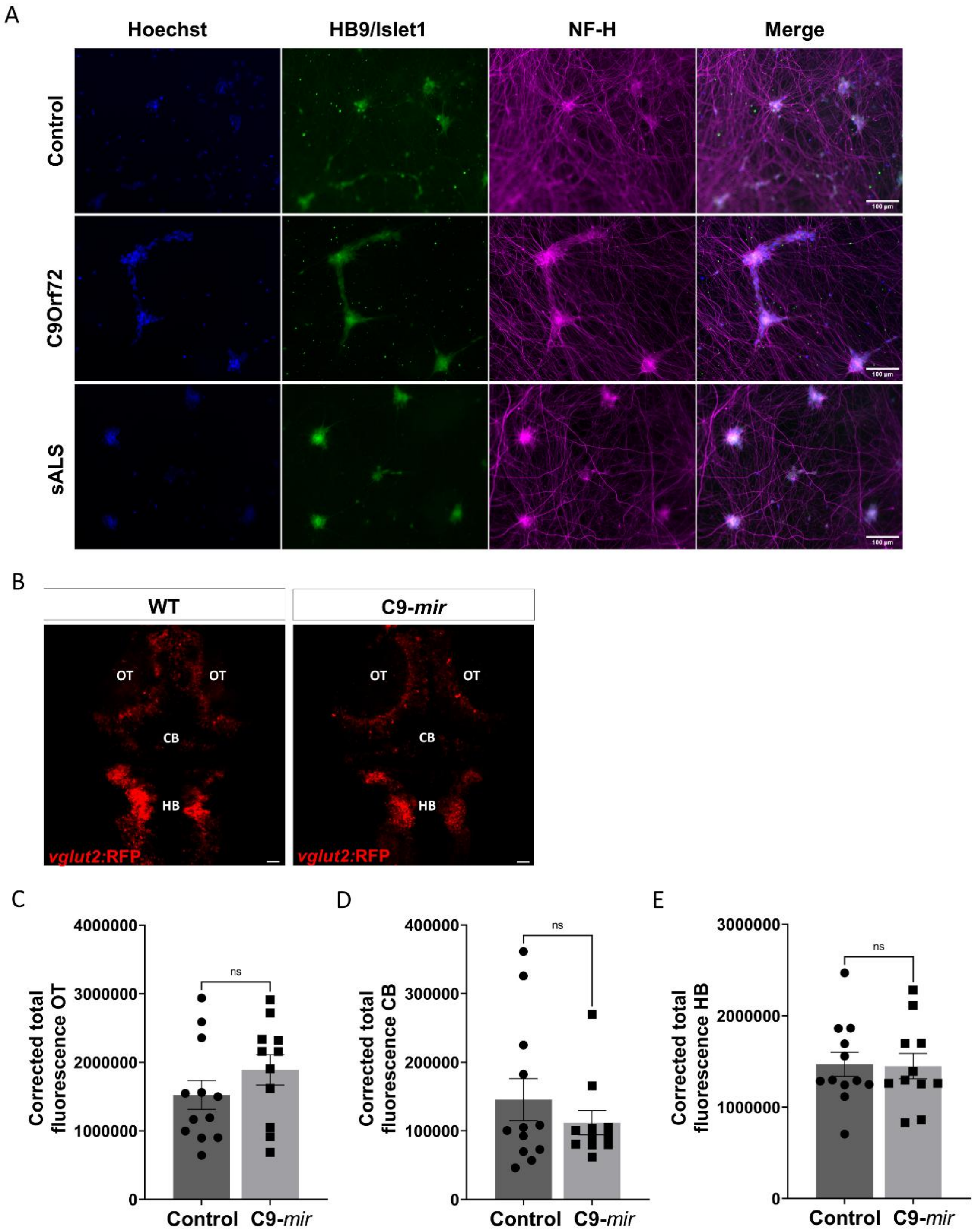

**Supplementary Fig. 1: Characterization of human iPSC-derived motor neurons and comparative analysis of glutamatergic neurons in control vs C9-*mir* zebrafish brain at 6 dpf**

A) Representative immunofluorescence images of iPSC-derived motor neurons. Cells were stained with the motor neuron-specific markers HB9 and Islet1 (HB9/ISL1) and the neuronal marker neurofilament heavy chain (NF-H). Nuclei were counterstained with Hoechst. Scale bar = 100 µm.

B) Representative images of glutamatergic neurons (*vglut2*:RFP) in control vs C9-*mir* zebrafish brain at 6 dpf. Scale bar = 20 µm.

C) Quantitative analysis demonstrated no significant changes in the corrected total fluorescence of Glutamatergic neurons (*vglut2*:RFP) in C9-*mir* zebrafish (1888410 ± 222049) compared to control zebrafish (1523668 ± 211304) optic tectum at 6 dpf, n=11-12.

D) Quantitative analysis demonstrated no significant changes in the corrected total fluorescence of Glutamatergic neurons (*vglut2*:RFP) in C9-*mir* zebrafish (111974 ± 17676) compared to control zebrafish (145565 ± 30548) cerebellum at 6 dpf, n= 11-12.

E) Quantitative analysis demonstrated no significant changes in the corrected total fluorescence of Glutamatergic neurons (*vglut2*:RFP) in C9-*mir* zebrafish (1448598 ± 138832) compared to control zebrafish (1468628 ± 131044) hind brain at 6 dpf, n= 11-12.

OT: Optic tectum; CB: Cerebellum; HB: Hindbrain

All data are represented as the mean ± standard error of the mean (SEM). Statistical significance was calculated by Student's t-test. ns : non-significant ; *P < 0.05; **P < 0.01; ***P < 0.001; ****P < 0.0001. n = sample size.

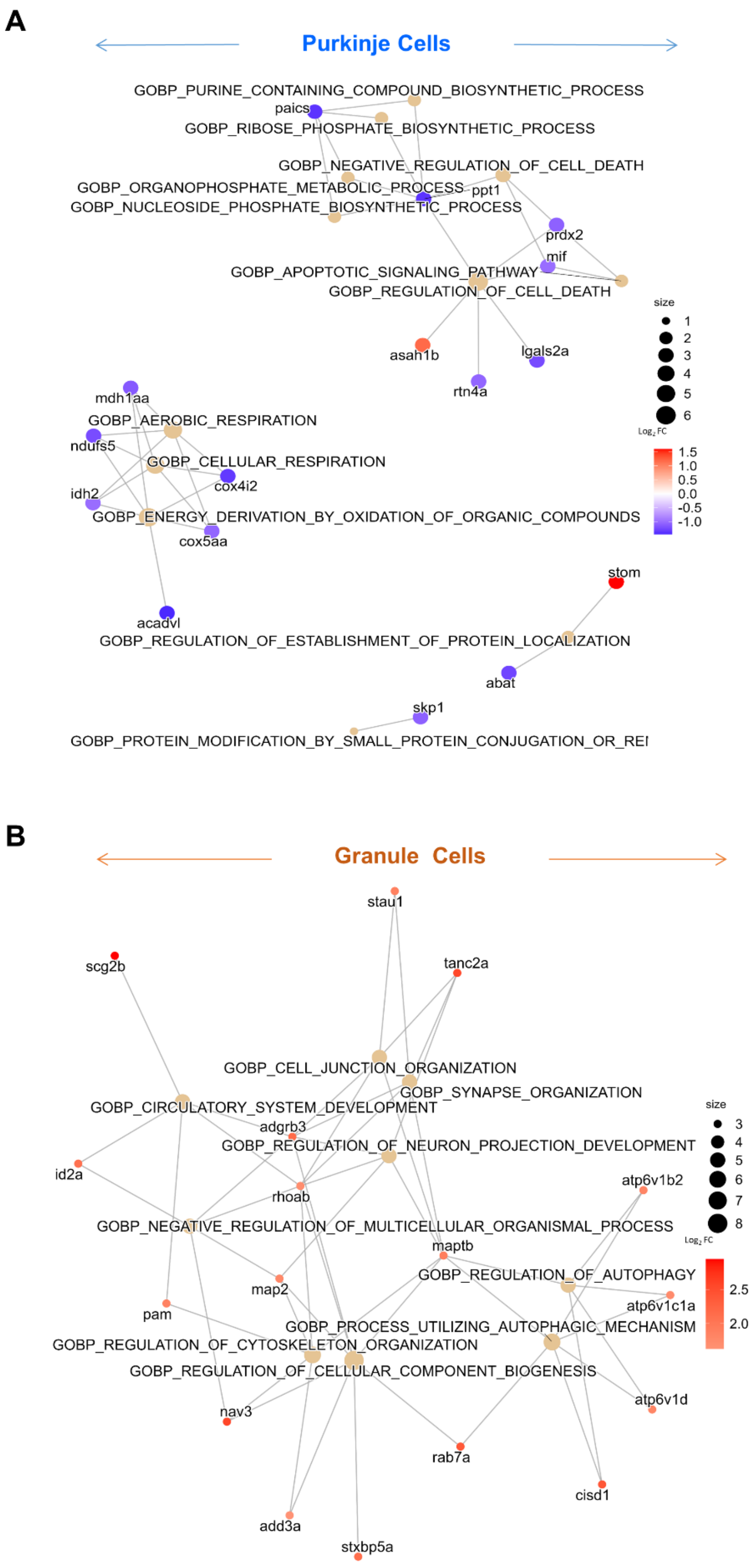
A
Purkinje Cells
GOBP_PURINE_CONTAINING_COMPOUND_BIOSYNTHETIC_PROCESS
paics
GOBP_RIBOSE_PHOSPHATE_BIOSYNTHETIC_PROCESS
GOBP_NEGATIVE_REGULATION_OF_CELL_DEATH
GOBP_ORGANOPHOSPHATE_METABOLIC_PROCESS
ppt1
GOBP_NUCLEOSIDE_PHOSPHATE_BIOSYNTHETIC_PROCESS
prdx2
mif
GOBP_APOPTOTIC_SIGNALING_PATHWAY
GOBP_REGULATION_OF_CELL_DEATH
asah1b
lgals2a
rtn4a
mdh1aa
GOBP_AEROBIC_RESPIRATION
ndufs5
GOBP_CELLULAR_RESPIRATION
cox4i2
idh2
GOBP_ENERGY_DERIVATION_BY_OXIDATION_OF_ORGANIC_COMPOUNDS
cox5aa
acadvl
stom
GOBP_REGULATION_OF_ESTABLISHMENT_OF_PROTEIN_LOCALIZATION
abat
skp1
GOBP_PROTEIN_MODIFICATION_BY_SMALL_PROTEIN_CONJUGATION_OR_REI
size
1
2
3
4
5
6
Log2 FC
1.5
1.0
0.5
0.0
-0.5
-1.0
B
Granule Cells
stau1
scg2b
tanc2a
GOBP_CELL_JUNCTION_ORGANIZATION
GOBP_SYNAPSE_ORGANIZATION
GOBP_CIRCULATORY_SYSTEM_DEVELOPMENT
adgrb3
GOBP_REGULATION_OF_NEURON_PROJECTION_DEVELOPMENT
id2a
rhoab
atp6v1b2
GOBP_NEGATIVE_REGULATION_OF_MULTICELLULAR_ORGANISMAL_PROCESS
maptb
map2
GOBP_REGULATION_OF_AUTOPHAGY
pam
atp6v1c1a
GOBP_PROCESS_UTILIZING_AUTOPHAGIC_MECHANISM
GOBP_REGULATION_OF_CYTOSKELETON_ORGANIZATION
GOBP_REGULATION_OF_CELLULAR_COMPONENT_BIOGENESIS
nav3
atp6v1d
rab7a
cisd1
add3a
stxbp5a
size
3
4
5
6
7
8
Log2 FC
2.5
2.0

**Supplementary Fig. 2: Gene set enrichment analysis reveals dysregulated biological processes in C9-*mir* cerebellar neurons.**

A-B) Cnet plot representing the linkage between the significantly dysregulated genes to enriched biological processes (BP) from gene set enrichment analysis (GSEA) in C9-*mir* (A) Purkinje cells and (B) Granule cells. The color gradient of the gene nodes indicates the direction and magnitude of the log2 fold change (red represents upregulation; blue represents downregulation).

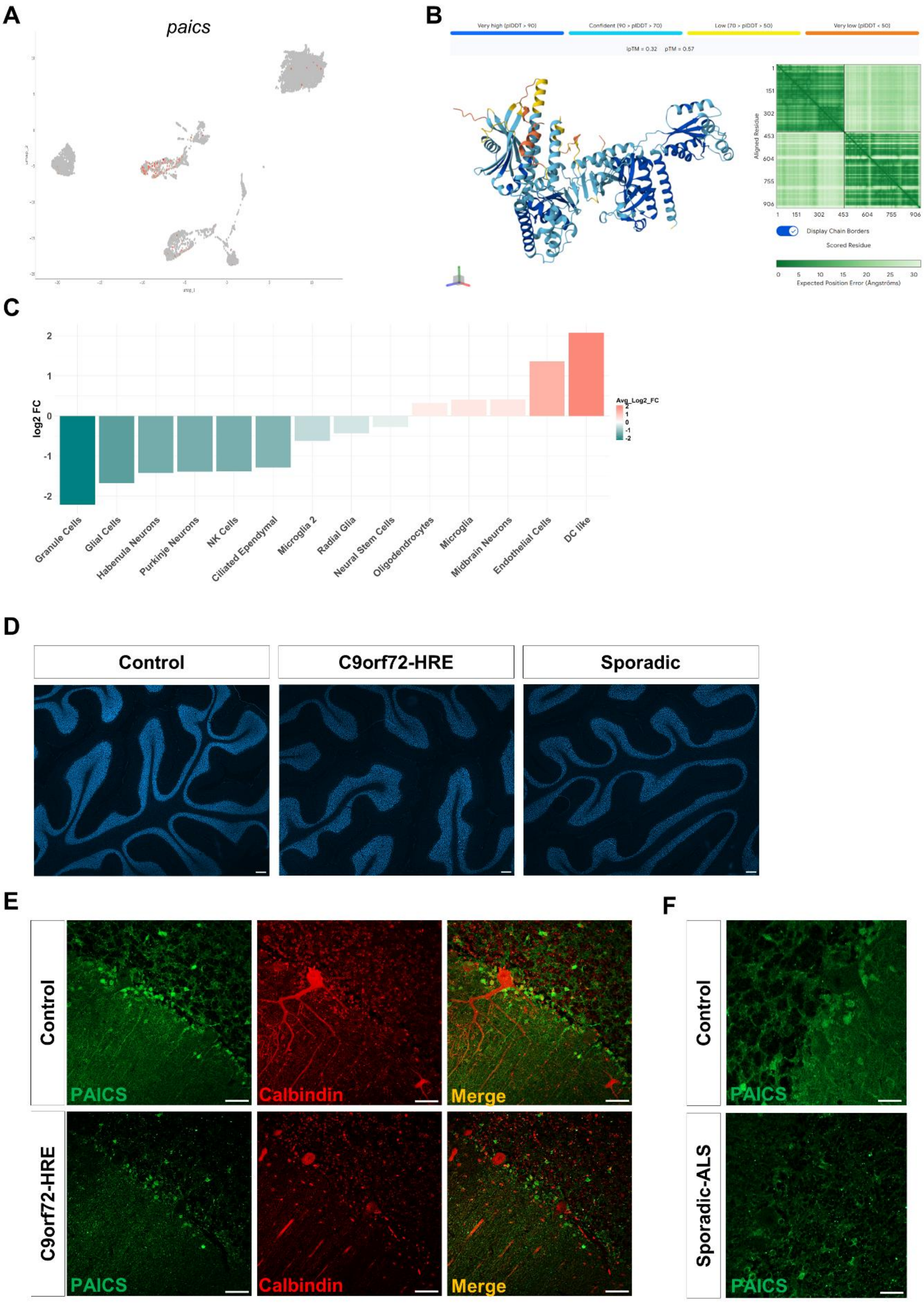
A
paics
B
Very high (pLDDT > 90)
Confident (90 > pLDDT > 70)
Low (70 > pLDDT > 50)
Very low (pLDDT < 50)
ipTM = 0.32 pTM = 0.57
Aligned Residue
1 151 302 453 604 755 906
Display Chain Borders
Scored Residue
0 5 10 15 20 25 30
Expected Position Error (Ångströms)
C
log2 FC
Avg_Log2_FC
Granule Cells
Glial Cells
Habenula Neurons
Purkinje Neurons
NK Cells
Ciliated Ependymal
Microglia 2
Radial Glia
Neural Stem Cells
Oligodendrocytes
Microglia
Midbrain Neurons
Endothelial Cells
DC like
D
Control
C9orf72-HRE
Sporadic
E
Control
C9orf72-HRE
PAICS
Calbindin
Merge
F
Control
Sporadic-ALS
PAICS

**Supplementary Fig. 3: PAICS expression in C9-*mir* zebrafish and post-mortem cerebellum samples**

A) The UMAP feature plots represent spatial expression patterns of *paics* across cell clusters.

B) The figure represents the AlphaFold 3 predicted PAICS-C9orf72 complex (left). The residues are coloured by pLDDT confidence score (blue = very high, sky-blue = confident, yellow = low, orange = very low). The corresponding predicted aligned error (PAE) heatmap (right) shows low intra-chain error but relatively high and diffuse inter-chain error. The model yields a low interface predicted TM-score (ipTM = 0.32) and moderate overall pTM (0.57), indicating limited confidence in a well-defined PAICS-C9orf72 complex.

C) Expression of the *paics* gene in C9-*mir* zebrafish brain across different cell types

D) Hoechst staining revealing the cerebellar foliation in control, *C9orf72*-HRE and sporadic post-mortem cerebellum sections.

E) PAICS (green) and Calbindin (Purkinje neurons, red) expression in post-mortem cerebellum section of control and *C9orf72*-HRE ALS patient.

F) PAICS expression in post-mortem cerebellum section of a control and a sporadic ALS patient.

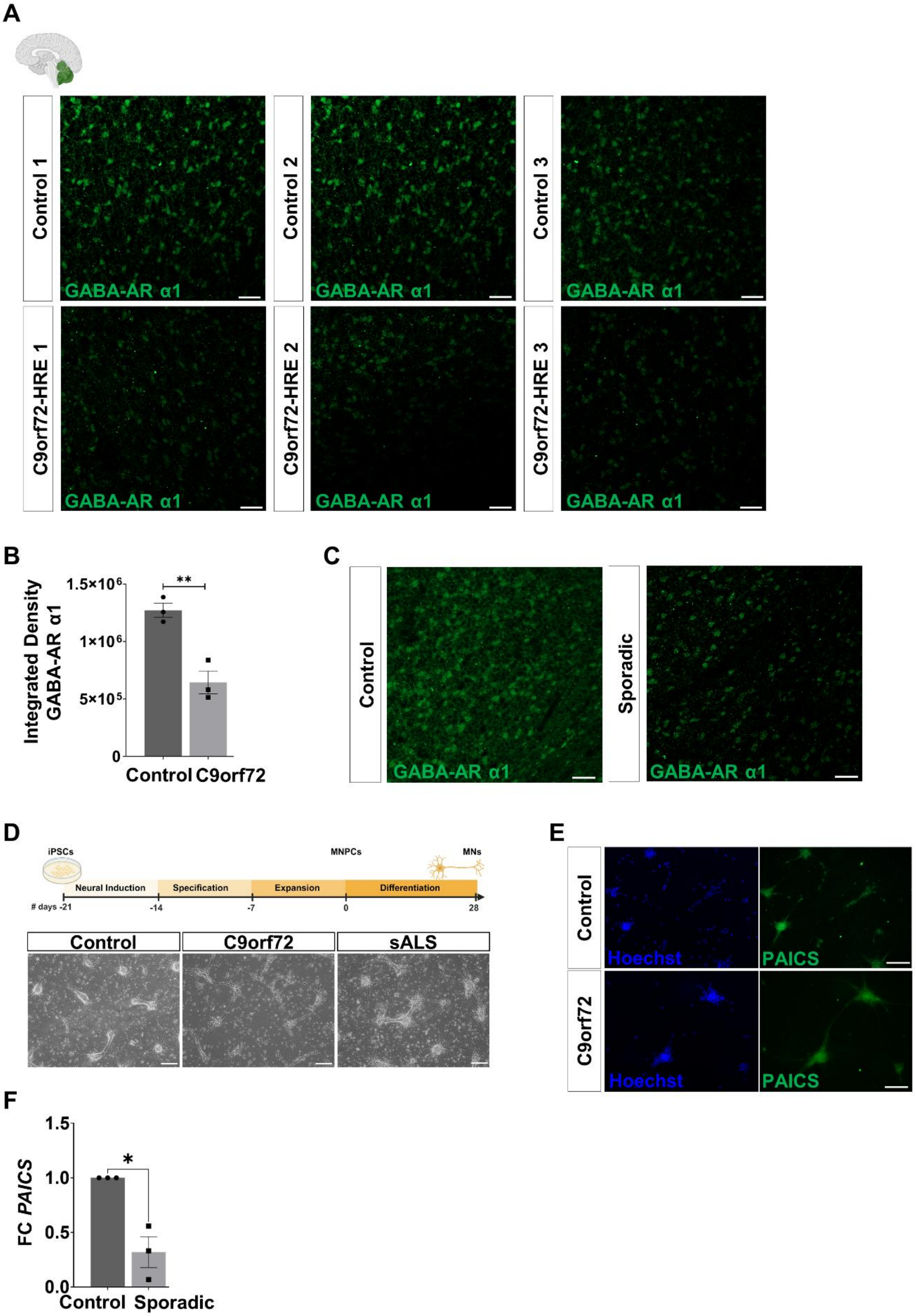
A
Control 1
Control 2
Control 3
GABA-AR α1
C9orf72-HRE 1
C9orf72-HRE 2
C9orf72-HRE 3
B
Integrated Density GABA-AR α1
1.5×10^6
1×10^6
5×10^5
0
**
Control
C9orf72
C
Control
Sporadic
GABA-AR α1
D
iPSCs
MNPCs
MNs
Neural Induction
Specification
Expansion
Differentiation
# days -21
-14
-7
0
28
Control
C9orf72
sALS
E
Control
C9orf72
Hoechst
PAICS
F
FC PAICS
1.5
1.0
0.5
0.0
*
Control
Sporadic

**Supplementary Fig. 4: Loss of granule cells in post-mortem cerebellum tissue of ALS patients and reduced *PAICS* expression in iPSC-MNs from sporadic ALS patients**

A) Immunofluorescence staining of GABA-AR α1 (Granule cells) in post-mortem cerebellar sections from control and *C9orf72*-HRE ALS patients. N=3. Scale bar = 30 µm.

B) Quantitative analysis demonstrated a significant decrease in GABA-AR α1 signal in *C9orf72*-HRE cerebellum sections (644146 ± 98803) compared to controls (1271685 ± 62193). (N=3).

C) Immunofluorescence staining of GABA-AR α1 (Granule cells) in post-mortem cerebellar section from a control and a sporadic ALS patient. Scale bar = 30 µm.

D) Schematic representation of the protocol for sequential differentiation of iPSCs into neuroepithelial progenitors (NEPs), motor neuron progenitor cells (MNPCs), and motor neurons (MNs). Representative phase-contrast images show the morphology of differentiated motor neurons after 3 weeks of differentiation. Scale bar = 100 µm.

E) Representative immunofluorescence images of iPSC-derived motor neurons from control and *C9orf72*-HRE patients. Cells were stained with PAICS (green), and nuclei were counterstained with Hoechst (blue). Scale bar = 100 µm.

F) Quantitative PCR (qPCR) analysis of *PAICS* mRNA in iPSC-derived motor neurons (MNs) from control and sporadic ALS patients at 3 week post-differentiation. (N'=3).

All data are represented as the mean ± standard error of the mean (SEM). Statistical significance was calculated by one-tailed Student's t-test (with Welch's correction for unequal variances). *P < 0.05; **P < 0.01; ***P < 0.001; ****P < 0.0001. N = number of human donors from whom postmortem cerebellar tissue was obtained; N'= number of donors from whom iPSC-MN lines were generated.

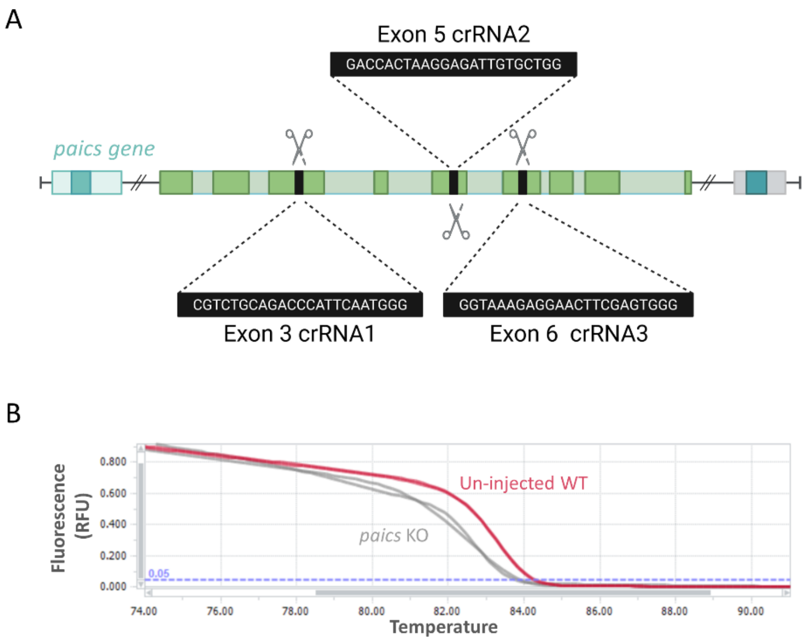


**Supplementary Fig. 5: Generation of *paics* knockout zebrafish line**

A) Schematic representation of the three crRNA target sites at exon 3, 5 and 6 of the zebrafish *paics* gene to create a *paics* F0 CRISPR zebrafish knockout (KO) line.

B) Genotyping of un-injected and RNP-injected zebrafish larvae using high-resolution melt curve analysis distinguishes between un-injected wild-type (WT) and *paics* F0 KO zebrafish.

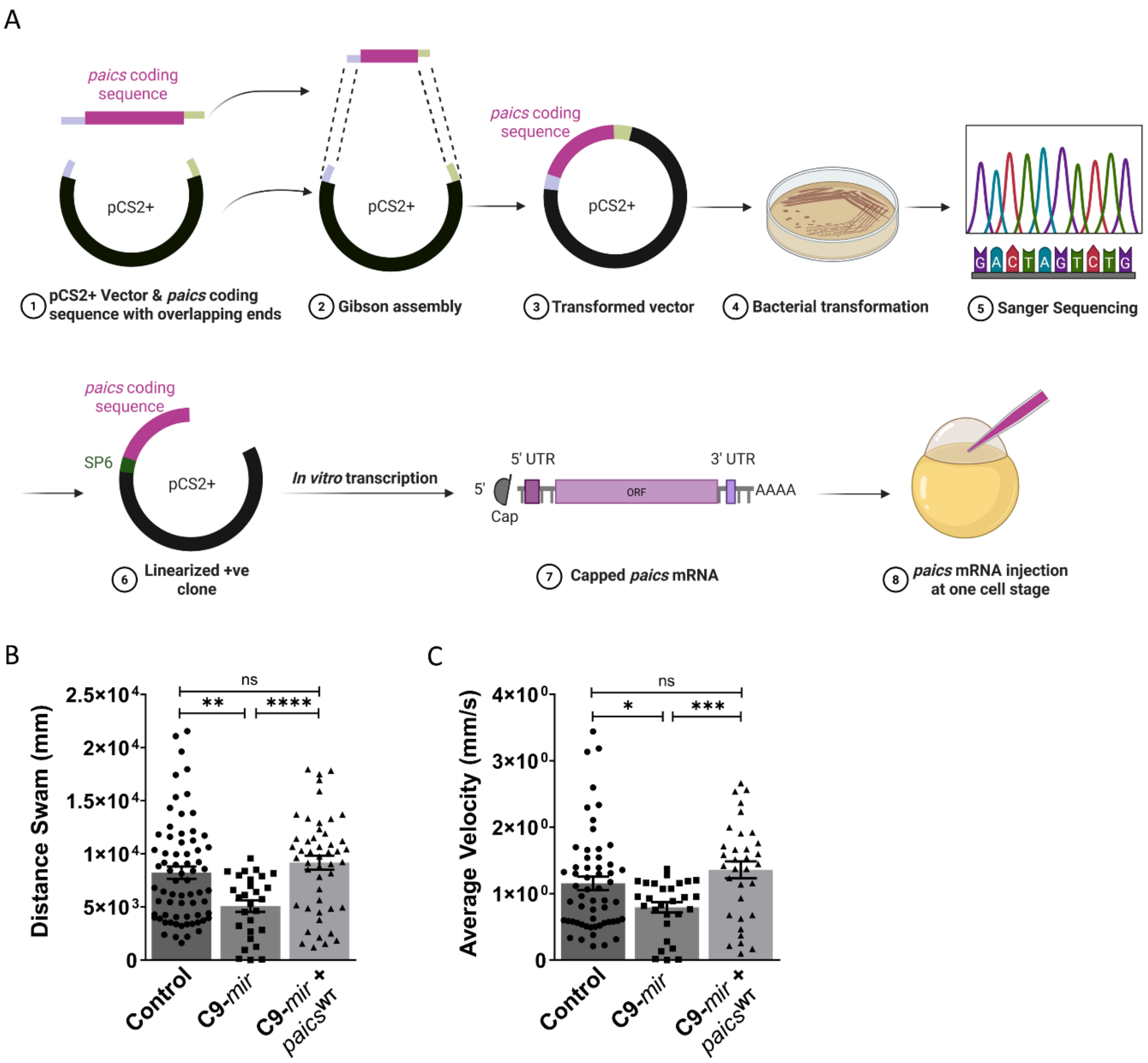


**Supplementary Fig. 6: Generation of capped *paics* mRNA and functional rescue of locomotor deficits in C9-*mir* zebrafish**

A) Illustration depicting the generation and injection of WT *paics* mRNA at one-cell stage zebrafish embryos.

B) Quantification of swimming distance and C) average velocity in control, C9-*mir* and C9-*mir* + *paics*$^{WT}$ zebrafish at 6 dpf.

All data are represented as the mean ± standard error of the mean (SEM). Statistical significance was calculated by one-way ANOVA followed by Dunnett's T3 multiple comparisons test. ns : non-significant ; *P < 0.05; **P < 0.01; ***P < 0.001; ****P < 0.0001. n = sample size.

## Supplementary Tables

| DEG in scRNA data of C9-*mir* zebrafish brain | Cell Type | Association of the gene with ALS in some publicly available studies |
|---|---|---|
| ***rtn4a*** | **PC** | **(Pradat et al., 2007 [6]; Rodriguez et al., 2022 [7]; Yang et al., 2009 [8])** |
| ***abat*** | **PC** | **(Farrawell et al., 2023 [9])** |
| ***maptb*** | **GC** | **(Fang et al., 2013 [10]; Origone et al. [11], 2018; Petrozziello et al., 2022 [12]; Udine E et al; 2024 [13] )** |
| ***atp6v0a1b*** | **GC** | **(Kim et al., 2022 [14])** |
| ***ctnnb1*** | **CE** | **(Hawkins et al., 2022 [15]; Lee et al., 2023 [16])** |
| ***sparc*** | **CE** | **(MacLean et al., 2022 [17])** |
| ***grn1*** | **DC-Like** | **(Cannon et al. [18], 2013; Nalls et al., 2021 [19])** |
| ***cd74a*** | **DC-Like** | **(Alfahel et al., 2024 [20]; Zhang & Sun, 2023 [21])** |
| ***ubc*** | **Eth Cells** | **(Farrawell et al., 2020, 2023 [9,22])** |
| ***selenop*** | **Glial Cells** | **(Donnelly et al., 2013 [23]; Urbano et al., 2022 [24])** |
| ***tsc22d3*** | **Glial Cells** | **(Donnelly et al., 2013 [23]; Ziye et al., 2023 [25])** |
| ***rtn1b*** | **HN** | **(Lederer et al., 2007 [26])** |
| ***ttbk1b*** | **HN** | **(Liachko et al., 2014 [27]; Nozal et al., 2022 [28]; Tian et al., 2021 [29])** |

| | | |
|---|---|---|
| ***lgals9l1*** | **MG** | **(Cai et al., 2022 [30])** |
| ***SERP1*** | **MG, MG2, NK Cells** | **(Selvaraj et al., 2018 [31])** |
| ***apoeb*** | **MG** | **(Lacomblez et al., 2002 [32]; Moulard et al., 1996 [33])** |
| ***snap25a*** | **MBN** | **(Yazar et al., 2023 [34])** |
| ***atp6v0e1*** | **NSC** | **(Kim et al., 2022 [14])** |
| ***b2ml*** | **NSC** | **(Staats et al., 2013 [35])** |
| ***s100b*** | **NSC** | **(Otto et al., 1998 [36])** |
| ***gpx4a*** | **Olg** | **(Chen et al., 2021 [37]; Tu et al., 2023 [38])** |
| ***pdcd4b*** | **Olg** | **(Li et al., 2020 [39])** |

**Supplementary Table 1: Significant scRNA-seq DEGs in C9-*mir* zebrafish brain cell types linked to ALS:** Significant DEGs found in scRNA data of C9-*mir* zebrafish brain in different cell types and their association/report in already published public studies of ALS. PC : Purkinje Cells, GC: Granule cells, CE : Ciliated Ependymal , DC : Dendritic Cell, Eth Cells : Endothelial Cells, HN : Habenula Neurons, MG : Microglia, MG2 : Microglia2, NK Cells : Natural Killer Cells, MBN : Mid Brain Neurons, NSC : Neural Stem Cells, Olg : Oligodendrocytes

| **Gene** | **Cell Type** | **log2FC** | **% of cells expressing (WT)** | **% of cells expressing (C9-*mir*)** |
|---|---|---|---|---|
| ***paics*** | **PC** | **-1.389** | **41.9** | **68.8** |

| | | | | |
|---|---|---|---|---|
| ***rtn4a*** | **PC** | **-1.067** | **54.8** | **87.5** |
| ***tanc2a*** | **GC** | **2.554** | **40** | **7.8** |
| ***maptb*** | **GC** | **1.924** | **65** | **20** |

**Supplementary Table 2: Top differentially expressed genes (DEGs) in cerebellar cell types of C9-*mir* zebrafish**: Log2 fold change (log2FC) and percentage of cells expressing each gene in WT and C9-*mir* zebrafish are shown. PCs: Purkinje cells; GCs: Granule cells. Data derived from single-cell RNA sequencing analysis.

# Author contributions

J.S. and S.A.P. conceived the present study. J.S. and L.L. designed the experiments. JS perfomed all zebrafish experiments, single cell RNA-seq data analysis, human post-mortem section staining and qPCR for human iPSC-MNs. L.L performed single cell RNA seq protocol and generated human iPSC-MN lines. C.Z assisted in the cloning. J.S. analyzed the data. T.D. provided access to his iPSC platform to generate ALS patient-derived motor neurons. M.C and G.H generated human iPSC-MN progenitors. J.S. wrote the manuscript. All authors reviewed and edited the manuscript. S.A.P. acquired funding and supervised the study.

---

# References


1. Measuring cell fluorescence using ImageJ — The Open Lab Book v1.0. Accessed January 2, 2025. https://theolb.readthedocs.io/en/latest/imaging/measuring-cell-fluorescence-using-imagej.html
2. Taylor SC, Rosselli-Murai LK, Crobeddu B, Plante I. A critical path to producing high quality, reproducible data from quantitative western blot experiments. *Scientific Reports 2022 12:1*. 2022;12(1):17599-. doi:10.1038/s41598-022-22294-x

3. Hao Y, Stuart T, Kowalski MH, et al. Dictionary learning for integrative, multimodal, and massively scalable single-cell analysis. *Nat Biotechnol*. 2023;42(2):293. doi:10.1038/S41587-023-01767-Y

4. McGinnis CS, Murrow LM, Gartner ZJ. DoubletFinder: Doublet Detection in Single-Cell RNA Sequencing Data Using Artificial Nearest Neighbors. *Cell Syst*. 2019;8(4):329-337.e4. doi:10.1016/j.cels.2019.03.003

5. Subramanian A, Tamayo P, Mootha VK, et al. Gene set enrichment analysis: A knowledge-based approach for interpreting genome-wide expression profiles. *Proc Natl Acad Sci U S A*. 2005;102(43):15545-15550. doi:10.1073/PNAS.0506580102/SUPPL_FILE/06580FIG7.JPG

6. Pradat PF, Bruneteau G, Gonzalez De Aguilar JL, et al. Muscle Nogo-A expression is a prognostic marker in lower motor neuron syndromes. *Ann Neurol*. 2007;62(1):15-20. doi:10.1002/ana.21122

7. Rodriguez CM, Bechek SC, Jones GL, et al. Targeting RTN4/NoGo-Receptor reduces levels of ALS protein ataxin-2. *Cell Rep*. 2022;41(4). doi:10.1016/j.celrep.2022.111505

8. Yang YS, Harel NY, Strittmatter SM. Reticulon-4A (Nogo-A) Redistributes Protein Disulfide Isomerase to Protect Mice from SOD1-Dependent Amyotrophic Lateral Sclerosis. *The Journal of Neuroscience*. 2009;29(44):13850. doi:10.1523/JNEUROSCI.2312-09.2009

9. Farrawell NE, Bax M, Mcalary L, et al. ALS-linked CCNF variant disrupts motor neuron ubiquitin homeostasis. *Hum Mol Genet*. 2023;32(14):2386. doi:10.1093/HMG/DDAD063

10. Fang P, Xu WY, Wu CS, Zhu M, Li XB, Hong DJ. MAPT as a predisposing gene for sporadic amyotrophic lateral sclerosis in the Chinese Han population. *Neural Regen Res*. 2013;8(33):3116. doi:10.3969/J.ISSN.1673-5374.2013.33.005

11. Origone P, Geroldi A, Lamp M, et al. Role of MAPT in Pure Motor Neuron Disease: Report of a Recurrent Mutation in Italian Patients. *Neurodegener Dis*. 2018;18(5-6):310-314. doi:10.1159/000497820

12. Petrozziello T, Amaral AC, Dujardin S, et al. Novel genetic variants in MAPT and alterations in tau phosphorylation in amyotrophic lateral sclerosis post-mortem motor cortex and cerebrospinal fluid. *Brain Pathol*. 2022;32(2). doi:10.1111/BPA.13035

13. Udine E, DeJesus-Hernandez M, Tian S, et al. Abundant transcriptomic alterations in the human cerebellum of patients with a C9orf72 repeat expansion. *Acta Neuropathol*. 2024;147(1). doi:10.1007/S00401-024-02720-2

14. Kim G, Nakayama L, Blum JA, et al. Genome-wide CRISPR screen reveals v-ATPase as a drug target to lower levels of ALS protein ataxin-2. *Cell Rep*. 2022;41(4). doi:10.1016/j.celrep.2022.111508

15. Hawkins S, Namboori SC, Tariq A, et al. Upregulation of β-catenin due to loss of miR-139 contributes to motor neuron death in amyotrophic lateral sclerosis. *Stem Cell Reports*. 2022;17(7):1650. doi:10.1016/J.STEMCR.2022.05.019

16. Lee A, Henderson R, Arachchige BJ, Robertson T, McCombe PA. Proteomic investigation of ALS motor cortex identifies known and novel pathogenetic mechanisms. *J Neurol Sci*. 2023;452:120753. doi:10.1016/J.JNS.2023.120753

17. MacLean M, López-Díez R, Vasquez C, Gugger PF, Schmidt AM. Neuronal–glial communication perturbations in murine SOD1G93A spinal cord. *Communications Biology 2022 5:1*. 2022;5(1):1-16. doi:10.1038/s42003-022-03128-y

18. Cannon A, Fujioka S, Rutherford NJ, et al. Clinicopathologic variability of the GRN A9D mutation, including amyotrophic lateral sclerosis. *Neurology*. 2013;80(19):1771. doi:10.1212/WNL.0B013E3182919059

19. Nalls MA, Blauwendraat C, Sargent L, et al. Evidence for GRN connecting multiple neurodegenerative diseases. *Brain Commun*. 2021;3(2). doi:10.1093/BRAINCOMMS/FCAB095

20. Alfahel L, Gschwendtberger T, Kozareva V, et al. Targeting low levels of MIF expression as a potential therapeutic strategy for ALS. *Cell Rep Med*. 2024;5(5). doi:10.1016/J.XCRM.2024.101546/ASSET/26EBAF5A-959F-40B7-AF5F-960AA9D898E8/MAIN.ASSETS/GR7.JPG

21. Zhang Z, Sun S. Linking ALS mutations to the disruption of the MHC-II pathway. *Proc Natl Acad Sci U S A*. 2023;120(43):e2315240120. doi:10.1073/PNAS.2315240120/ASSET/C46D1D44-94B0-4ADF-8A64-B5010A9E7514/ASSETS/IMAGES/LARGE/PNAS.2315240120FIG01.JPG

22. Farrawell NE, McAlary L, Lum JS, et al. Ubiquitin Homeostasis Is Disrupted in TDP-43 and FUS Cell Models of ALS. *iScience*. 2020;23(11). doi:10.1016/J.ISCI.2020.101700

23. Donnelly CJ, Zhang PW, Pham JT, et al. RNA Toxicity from the ALS/FTD C9ORF72 Expansion Is Mitigated by Antisense Intervention. *Neuron*. 2013;80(2):415-428. doi:10.1016/J.NEURON.2013.10.015/ATTACHMENT/90740792-5095-427E-BDB7-C6FA688FF696/MMC14.XLSX

24. Urbano T, Vinceti M, Mandrioli J, et al. Selenoprotein P Concentrations in the Cerebrospinal Fluid and Serum of Individuals Affected by Amyotrophic Lateral Sclerosis, Mild Cognitive Impairment and Alzheimer's Dementia. *Int J Mol Sci*. 2022;23(17). doi:10.3390/IJMS23179865/S1

25. Ziye A, 1# M, Kirk BD, et al. Single-cell transcriptional landscape of peripheral immunity and biomarkers for human sporadic amyotrophic lateral sclerosis patients. *medRxiv*. Published online November 1, 2023:2022.07.03.22277129. doi:10.1101/2022.07.03.22277129

26. Lederer CW, Torrisi A, Pantelidou M, Santama N, Cavallaro S. Pathways and genes differentially expressed in the motor cortex of patients with sporadic amyotrophic lateral sclerosis. *BMC Genomics*. 2007;8(1):1-26. doi:10.1186/1471-2164-8-26/FIGURES/7

27. Liachko NF, McMillan PJ, Strovas TJ, et al. The Tau Tubulin Kinases TTBK1/2 Promote Accumulation of Pathological TDP-43. *PLoS Genet*. 2014;10(12):1004803. doi:10.1371/JOURNAL.PGEN.1004803

28. Nozal V, Martínez-González L, Gomez-Almeria M, et al. TDP-43 Modulation by Tau-Tubulin Kinase 1 Inhibitors: A New Avenue for Future Amyotrophic Lateral Sclerosis Therapy. *J Med Chem*. 2022;65(2):1585-1607. doi:10.1021/ACS.JMEDCHEM.1C01942/SUPPL_FILE/JM1C01942_SI_002.CSV

29. Tian Y, Wang Y, Jablonski AM, et al. Tau-tubulin kinase 1 phosphorylates TDP-43 at disease-relevant sites and exacerbates TDP-43 pathology. *Neurobiol Dis*. 2021;161. doi:10.1016/J.NBD.2021.105548

30. Cai Z, Yin K, Liu Q, Liu M, Yang X, Cui L. Association between abnormal expression and methylation of LGALS1 in amyotrophic lateral sclerosis. *Brain Res*. 2022;1792:148022. doi:10.1016/J.BRAINRES.2022.148022

31. Selvaraj BT, Livesey MR, Zhao C, et al. C9ORF72 repeat expansion causes vulnerability of motor neurons to Ca2+-permeable AMPA receptor-mediated excitotoxicity. *Nat Commun*. 2018;9(1). doi:10.1038/S41467-017-02729-0

32. Lacomblez L, Doppler V, Beucler I, et al. APOE: A potential marker of disease progression in ALS. *Neurology*. 2002;58(7):1112-1114. doi:10.1212/WNL.58.7.1112

33. Moulard B, Sefiani A, Laamri A, Malafosse A, Camu W. Apolipoprotein E genotyping in sporadic amyotrophic lateral sclerosis: evidence for a major influence on the clinical presentation and prognosis. *J Neurol Sci*. 1996;139 Suppl(SUPPL.):34-37. doi:10.1016/0022-510X(96)00085-8

34. Yazar V, Kühlwein JK, Knehr A, et al. Impaired ATF3 signaling involves SNAP25 in SOD1 mutant ALS patients. *Scientific Reports 2023 13:1*. 2023;13(1):1-13. doi:10.1038/s41598-023-38684-8

35. Staats KA, Schönefeldt S, Van Rillaer M, et al. Beta-2 microglobulin is important for disease progression in a murine model for amyotrophic lateral sclerosis. *Front Cell Neurosci*. 2013;7:67230. doi:10.3389/FNCEL.2013.00249

36. Otto M, Bahn E, Wiltfang J, Boekhoff I, Beuche W. Decrease of S100 beta protein in serum of patients with amyotrophic lateral sclerosis. *Neurosci Lett*. 1998;240(3):171-173. doi:10.1016/S0304-3940(97)00947-6

37. Chen L, Na R, Danae McLane K, et al. Overexpression of ferroptosis defense enzyme Gpx4 retards motor neuron disease of SOD1G93A mice. *Scientific Reports 2021 11:1*. 2021;11(1):1-13. doi:10.1038/s41598-021-92369-8

38. Tu LF, Zhang TZ, Zhou YF, et al. GPX4 deficiency-dependent phospholipid peroxidation drives motor deficits of ALS. *J Adv Res*. 2023;43:205. doi:10.1016/J.JARE.2022.02.016

39. Li C, Chen Y, Chen X, et al. MicroRNA-183-5p is stress-inducible and protects neurons against cell death in amyotrophic lateral sclerosis. *J Cell Mol Med*. 2020;24(15):8614-8622. doi:10.1111/JCMM.15490

# Raw Western Blot Images

A

**Anti-PAICS antibody**

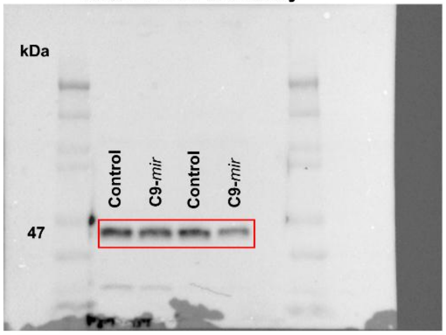


Merged blot with ladder; refer to Fig. 4B-C

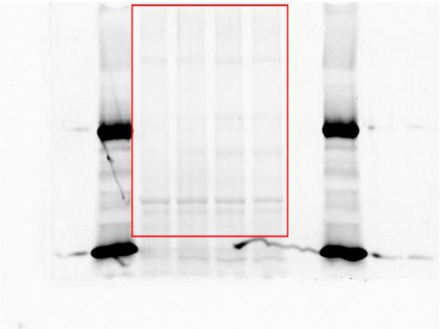

Total Protein; refer to Fig. 4B-C

**Anti-PAICS antibody**

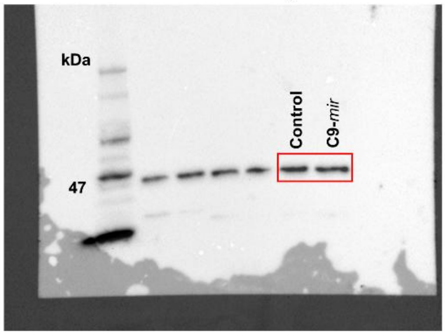


Merged blot with ladder; refer to Fig. 4C

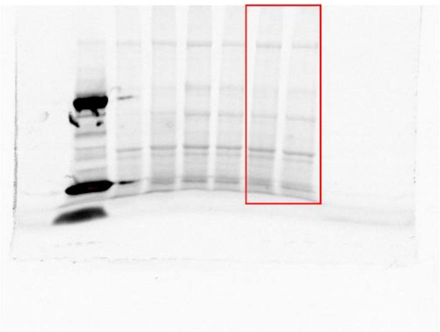

Total Protein; refer to Fig. 4C

***Zebrafish***

B

**Anti-PAICS antibody**

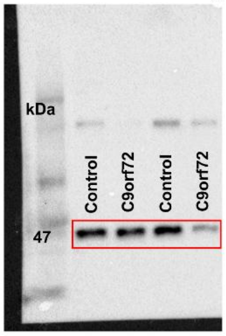


Merged blot with ladder; refer to Fig. 4G-H

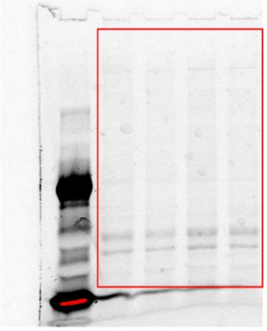

Total Protein; refer to Fig. 4G-H

**Anti-PAICS antibody**

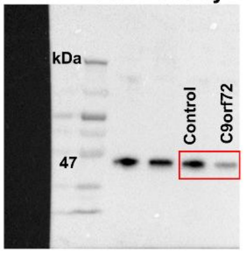


Merged blot with ladder; refer to Fig. 4H

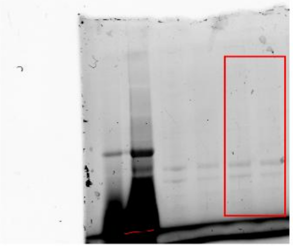

Total Protein; refer to Fig. 4H

***iPSC-MNs***
